# SIGIRR Downregulation and Interleukin-1 Signaling Intrinsic to Renal Cell Carcinoma

**DOI:** 10.3389/fonc.2022.894413

**Published:** 2022-06-22

**Authors:** Maria Elena Mantione, Ilenia Sana, Maria Giovanna Vilia, Michela Riba, Claudio Doglioni, Alessandro Larcher, Umberto Capitanio, Marta Muzio

**Affiliations:** ^1^ Cell Signaling Unit, Division of Experimental Oncology, San Raffaele Hospital Istituto di Ricovero e Cura a Carattere Scientifico (IRCCS), Milano, Italy; ^2^ Center for Omics Sciences, San Raffaele Hospital Istituto di Ricovero e Cura a Carattere Scientifico (IRCCS), Milano, Italy; ^3^ Pathology Unit, San Raffaele Hospital Istituto di Ricovero e Cura a Carattere Scientifico (IRCCS), Milano, Italy; ^4^ Department of Urology, San Raffaele Scientific Institute, Milan, Italy; Division of Experimental Oncology/Unit of Urology, Urological Research Institute (URI), Istituto di Ricovero e Cura a Carattere Scientifico (IRCCS) San Raffaele Hospital, Milan, Italy

**Keywords:** SIGIRR, renal cell carcinoma, PDL1, interleukin-1, NFKBIZ, inflammation

## Abstract

Renal cell carcinoma is highly inflamed, and tumor cells are embedded into a microenvironment enriched with IL1. While inflammatory pathways are well characterized in the immune system, less is known about these same pathways in epithelial cells; it is unclear if and how innate immune signals directly impact on cancer cells, and if we could we manipulate these for therapeutic purposes. To address these questions, we first focused on the inflammatory receptors belonging to the IL1- and Toll-like receptor family including negative regulators in a small cohort of 12 clear cell RCC (ccRCC) patients’ samples as compared to their coupled adjacent normal tissues. Our data demonstrated that renal epithelial cancer cells showed a specific and distinctive pattern of inflammatory receptor expression marked by a consistent downregulation of the inhibitory receptor SIGIRR mRNA. This repression was confirmed at the protein level in both cancer cell lines and primary tissues. When we analyzed *in silico* data of different kidney cancer histotypes, we identified the clear cell subtype as the one where SIGIRR was mostly downregulated; nonetheless, papillary and chromophobe tumor types also showed low levels as compared to their normal counterpart. RNA-sequencing analysis demonstrated that IL1 stimulation of the ccRCC cell line A498 triggered an intrinsic signature of inflammatory pathway activation characterized by the induction of distinct “pro-tumor” genes including several chemokines, the autocrine growth factor IL6, the atypical co-transcription factor NFKBIZ, and the checkpoint inhibitor PD-L1. When we looked for the macroareas most represented among the differentially expressed genes, additional clusters emerged including pathways involved in cell differentiation, angiogenesis, and wound healing. To note, SIGIRR overexpression in A498 cells dampened IL1 signaling as assessed by a reduced induction of NFKBIZ. Our results suggest that SIGIRR downregulation unleashes IL1 signaling intrinsic to tumor cells and that manipulating this pathway may be beneficial in ccRCC.

## Introduction

Kidney cancer includes distinct histotypes of renal cell carcinoma (RCC) originating from epithelial cells lining the tubules. Clear cell RCC is the most common followed by two distinct papillary subtypes and chromophobe tumors (1). Even if RCC does not have a high tumor mutation burden relative to other cancers, it is an immunogenic tumor and the use of novel immunotherapy approaches including checkpoint inhibitors is now the standard strategy in the metastatic and adjuvant settings (2–4). Nevertheless, drug resistance or clinical unresponse may still occur in a non-negligible proportion of patients, raising the evidence that additional pathways yet to be fully characterized may play a role in this context (5). Moreover, prediction of recurrence, progression, and cancer-specific mortality in RCC is still based on clinical parameters only and potential molecular predictors are still waited (6).

RCC is an inflamed tumor with high infiltration of immune cells (7), and several inflammation-based prognostic scores predict adverse clinical outcomes (8–15). Inflammatory cytokines such as IL1β and IL6, chemokines, and matrix metalloproteinases as well as a variety of inflammatory transcription factors are upregulated and activated in patients affected by RCC, both systemically and inside the tumor site (16–23). Immune infiltration correlates with poor outcome but unfortunately does not help to precisely predict immunotherapy efficacy that is regulated by complex mechanisms currently under investigation (4, 5, 24–26).

Molecular studies revealed that infiltrating macrophages secrete inflammatory cytokines and blocking IL1β reduced the pro-tumoral effect of these cells in preclinical models of RCC (21); more recently, a multidimensional analysis suggested that immunotherapy combined with inhibition of IL1β promoted tumor regression and remodeled the immune infiltration in mice (27). Moreover, an elevated expression of IL1β was detected in RCC patients’ plasma and circulating monocytes; higher levels of this cytokine inside the tumor correlated with advanced tumor stage as well as with macrophage infiltration (18, 19, 21).

Overall, all these data confirm that tumor cells are embedded into an inflammatory microenvironment enriched with IL1 and that targeting this pathway may represent a promising therapeutic strategy especially in combination with immune checkpoint blockade. However, while inflammatory pathways are very well characterized in the immune system, less is known about these same pathways in epithelial cells; it is unclear if and how inflammatory signals directly impact on cancer cells, and several questions are emerging: Do cancer cells express specific inflammatory receptors? How do RCC cells respond to IL1? Can we manipulate this signaling pathway?

To address these questions, we focused our attention onto the inflammatory receptors belonging to the IL1- and Toll-like receptor family including negative regulators such as SIGIRR (Single ImmunoGlobulin Interleukin-1 Receptor-Related molecule; also known as TIR8 or interleukin-1 receptor 8) (28, 29). In addition to its well-known anti-inflammatory properties, the tumor-suppressive functions of SIGIRR have been recently described in intestinal cancer and chronic lymphocytic leukemia where SIGIRR is specifically downregulated both at mRNA and protein levels (30, 31). Quite the opposite in breast cancer, SIGIRR is upregulated and promotes tumor growth (32). SIGIRR mRNA is highly expressed in kidney epithelial cells (33), but no data are available about its expression and function in RCC.

We aimed at characterizing the expression pattern and functional role of SIGIRR as well as other inflammatory receptors intrinsic to renal cancer cells following the hypothesis that this class of prototypic immune receptors may directly regulate the pathobiology of solid tumors when associated with inflammation.

## Materials and Methods

### Patient Samples

Patients submitted to surgery provided respectively normal kidney tissue and tumor tissue. All patients signed an informed consent, and the study protocol conformed to the ethical guidelines of the Declaration of Helsinki and was approved by the ethics committee at IRCCS San Raffaele Hospital. All tissue samples were obtained with the approval of the Institutional Ethics Committee of San Raffaele Scientific Institute (Milan, Italy), after informed consent (Protocol RENE-2007 and URBBAN-2010). A portion of the tissue was frozen in liquid nitrogen for histochemistry (performed by Pathology Unit) and isolation of RNA and proteins.

### Cell Lines and Treatments

Renal cancer cell lines (Caki1, Caki2, A498, ACHN) were purchased from the American Type Culture Collection (ATCC) and were cultured in RPMI supplemented with 10% heat-inactivated FBS and 1% penicillin/streptomycin, in a standard incubator (37°C, 5% CO_2_, normoxia). Cells were treated with increasing concentrations of human IL1β (10–30–100 ng/ml) for 4 h (cat. 200-01B PeproTech, Rocky Hill, NJ). Cell lines were screened with a multiplex PCR method that provides a unique DNA fingerprint for each individual cell line, using the QIAGEN multiplex PCR kit following the manufacturer’s instruction.

### Immunofluorescence and Immunohistochemistry

Cells were fixed in 4% paraformaldehyde and permeabilized in 0.1% Triton X-100 in PBS for 10 min, blocked in BSA 2% for 1 h, washed in PBS, and incubated overnight at 4°C with anti-NFκBiz diluted in 0.1% BSA (rabbit polyclonal, 1:100, cat. 9244 Cell Signaling, Danvers, MA, USA), followed by a 1-h incubation with the secondary antibody Alexa Fluor^®^ 488 IgG (H+L) conjugate (1:500, cat. A21200, Invitrogen, Carlsbad, CA, USA). Golgi apparatuses were stained with Golgin-97 antibody (monoclonal antibody, 1:200, cat. A21270, Invitrogen) overnight at 4°C and then labeled with a secondary antibody Alexa Fluor^®^ 594 conjugate (1:500, cat. A21203, Invitrogen) for 1 h at RT. Cell nuclei were stained with DAPI. After washing with PBS, cells were mounted in ProLong^®^ Gold Antifade Reagent (Life Technologies, Carlsbad, CA, USA) and analyzed with Leica Confocal SP8 in the Advanced Light and Electron Microscopy BioImaging Center (ALEMBIC). Immunohistochemistry (IHC) was performed by the Pathology Unit at IRCCS San Raffaele Hospital using an antibody specific for a long isoform of human SIGIRR on normal kidney tissues and clear cell RCC (ccRCC) tissues.

### Transfection

A498 cells at 60%–80% confluency were transiently transfected with a SIGIRR-L1 construct previously described (34) containing a Myc-DDK-Flag tag in the pUno expression vector (InvivoGen, San Diego, CA, USA), or with the pUno empty vector using jetOPTIMUS^®^ DNA Transfection Reagent following the manufacturer’s instructions (Polyplus-transfection ref.117-07). The complete medium containing the transfection mix was replaced after 8 h with complete fresh medium. After 16 h, cells were treated with IL1β for 4 h then harvested, and the pellets were used for RNA and protein extraction.

### Real-Time PCR

Total RNA was extracted from frozen tissues by using the Maxwell^®^ 16 LEV simply RNA Tissue Kit and Maxwell Instrument (Promega, Madison, WI, USA) quantified with NanoDrop and retro-transcribed with Random Primers. Real-time PCR was performed by using the following TaqMan assays: SIGIRR: Hs01546500_g1, IL1R1: Hs00991010_m1. Real-time PCR data were analyzed by the 2(-ΔCt) method and normalized to 18S (qHsaCEP0049956, Bio-Rad, Hercules, CA, USA).

RCC cell lines’ and N2 normal kidney cells’ (35) total RNA was extracted using RNasi Plus Mini Kit (cat. 74134, QIAGEN, Hilden, Germany) according to the manufacturer’s instructions. First-strand cDNA was synthesized using 1 μg total RNA with iScript Advanced cDNA RT Kit (cat.1725038, Bio-Rad). To detect mRNA levels of target gene expression, quantitative RT-PCR was performed using SsoAdvanced™ Universal Probe Master Mix (Bio-Rad) and the following Prime PCR probe assay (FAM or HeX dye labeled, cat.12001950, Bio-Rad): NFKBIZ (qHsaCEP0052487), SIGIRR (qHsaCEP0057555), β-actin (qHsaCEP0036280), ICAM1 (qHsaCEP0024986), GAPDH (qHsaCEP0041396), SIGIRR (qHsaCEP0057555), MMP1 (qHsaCEP0055366) or Applied Biosystems’ assays-on-demand IL6 (Hs00985639) and MMP9 (Hs00957562) (Cat. 4331182, Thermo Fisher Scientific, Waltham, MA, USA). For PDL-1, the following primers were used: FW: CGACTACAAGCGAATTACTG; RV: CTGCTTGTCCAGATGACTTC for β-actin: qHsaCED0036269.

The relative mRNA expression was calculated using 2(-ΔCt) or 2(-ΔΔCt) method, using GAPDH or β-actin or both as the endogenous reference gene, as indicated in the figure legends. Real-time PCR was performed using the CFX Connect Real-Time PCR Detection System with Probe protocol (Bio-Rad).

### TLR Pathway Expression Profiling With qRT-PCR Arrays

Gene expression profiling of the TLR pathway was performed by using a real-time PCR array (Prime PCR array “TLR signaling pathway,” SAB target list H96 cod 10034251, Bio-Rad Laboratories, Hercules, CA, USA) in 12 RCC patient samples matched with normal adjacent renal tissue samples according to the manufacturer’s instruction. We analyzed the expression of 84 key genes involved in the TLR pathway plus seven housekeeping genes (B2M, HPRT1, RPLP0, GAPDH, and ACTB, TBP) and specific gDNA and quality controls.

The difference between the Ct value of each gene of interest and the average Ct value of housekeeping genes in each sample was then measured as previously described (Fonte, et al., 2013) using 38 as upper Ct. Fold change in gene expression between different subgroups of patients was calculated by the 2(-ΔCt) algorithm. The relative mRNA expression levels of differentially expressed genes were reported only if (I) the fold difference (FD) in average 2(-ΔCt) tumor/2(-ΔCt) normal values was >2 or <0.5 (indicative of up- or downregulation, respectively) and (ii) the difference in 2(-ΔCt) values was found statistically significant (p < 0.01) after the paired t-test.

Gene expression profiling of the TLR pathway in RCC cell lines was performed as described above and analyzed with Bio-Rad CFX Maestro™ Software, creating a Gene Study to compare gene expression data from one or more real-time PCR runs using an inter-run calibrator to normalize between the runs.

### RNA Sequencing Analyses

RNA-Seq differential gene expression between cell lines treated with IL1β and untreated A498 ccRCC cell lines, through four different experimental pairs, was performed in Genomics Laboratory of CORS, Center for Omics Science, Ospedale San Raffaele. Sequencing was performed using Illumina NovaSeq S2 Xp.

The trimmed sequences obtained have been aligned to the human “Hg38” genome. Gene expression analysis was performed comparing treated samples toward controls and directly between two treatments. Differential gene expression analysis was done using limma (36). Genes were considered as “differentially expressed” if they satisfy both the following conditions: nominal p-value < 0.01 and absolute value of log2 fold change >1.

Further data analyses were performed using the Enrichr online tool using default parameters (https://maayanlab.cloud/Enrichr/) and presenting tabular data sorted for combination score and p-value >0.005 (37–39). Gene set enrichment analysis and leading-edge analysis were performed with GSEA software 4.2 using default parameters (http://software.broadinstitute.org/gsea). Intersections were presented with a Venn diagram and created with InteractiVenn [www.interactivenn.net (40)]. Enrichment map visualization and annotation of the network were performed with Cytoscape 3.9 and an enrichment map, the AutoAnnotate app (http://apps.cytoscape.org/).

### Western Blot Analysis

Cells were lysed in RIPA buffer supplemented with protease and phosphatase inhibitors. Protein samples were loaded on SDS-PAGE electrophoresis gel and then transferred to a nitrocellulose membrane with a dry system Trans-Blot Turbo Transfer system (Bio-Rad). The membranes were incubated overnight at 4°C with the appropriate antibody diluted at the optimal concentration in 5% (w/v) skimmed milk or BSA. Antibodies used for Western blot analysis were as follows: anti-FLAG M2 (cat. 05-826 mouse 1:1000 Millipore, Burlington, MA, USA), NFKBIZ (cat. 9244, rabbit, 1:1,000 Cell Signaling Technology), SIGIRR (Santa Cruz, cat. 271864 (A-4), mouse, 1:200, Dallas, TX, USA), and anti-β-actin HRP-conjugated (a3854 Merck Millipore 1:20,000). The blots were then washed in PBS with Tween 0.2% (PBST) and incubated with an appropriate secondary antibody HRP conjugated for 1 h at room temperature. The proteins were visualized using Clarity Western ECL Substrate (cat 1705060 Bio-Rad) and detected by ChemiDoc Imaging System (Bio-Rad). Densitometric analysis of protein bands from Western blot was performed with Image Lab Software 6.1 (Bio-Rad).

### Statistical Analysis

Statistical analyses were performed with GraphPad Prism Version 9.3 (La Jolla, CA, USA). Probability values of <0.05 were considered statistically significant as indicated in each figure legend. To compare two groups of samples, Mann–Whitney (unpaired) or Wilcoxon (paired) test was performed considering a 95% confidence interval (CI) with an alpha error of 0.05. For more than two groups, Kruskal (unpaired samples) or Friedman (paired samples) test followed by Dunnett’s multiple-comparison posttest was used to compare each of the treatment groups with control. For more than two groups and subgroups, two-way ANOVA was performed followed by Sidak multiple-comparison posttest. Both tests were performed computing a 95% CI with an alpha error of 0.05. Kaplan–Meier survival curve analysis was done using gene expression data of RCC patients (n = 441 from renal cell carcinoma, TCGA, Pan Cancer Atlas) based on the expression level of SIGIRR mRNA patients which were divided into “low-expression” (SIGIRR expression below the 25th percentile) and “high expression” (above the 75th percentile). The Kaplan–Meier estimate was used to plot survival, and statistical significance was assessed with the log-rank Mantel–Cox test with 95% CI.

## Results

### Inflammatory Receptor Expression Pattern in Kidney Cancer

**Figure 1:**
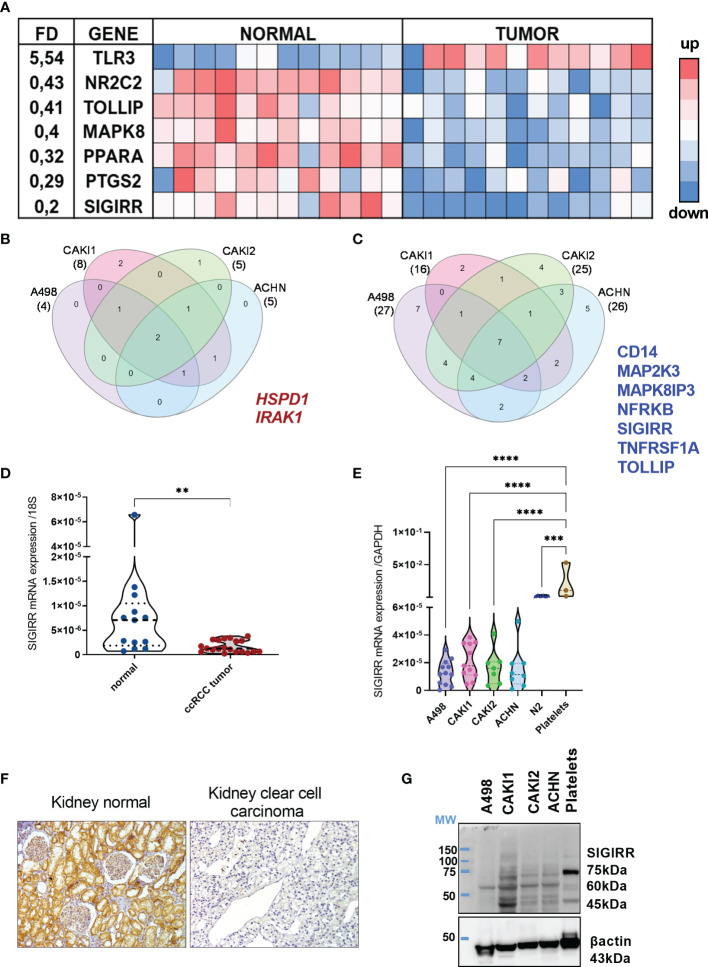
Immune signaling molecules in normal versus tumor kidney. **(A)** TLR signaling pathway-related molecules were analysis using real-time PCR arrays. Eighty-four genes listed in **Supplementary Table 1** were screened in 12 patients’ samples including coupled normal and malignant tissues. Heat map shows the genes differentially expressed (i.e., with a fold difference (FD) FD >2.0 or FD <0,5 and p-value <0.01). A three-color formatting scheme was used with blue indicating downregulated and red upregulated. **(B, C)** RCC cell lines were analyzed by real-time PCR array (same as in **(A)**) and compared to N2 normal renal cell lines to identify the genes up- and down-regulated; Venn diagram shows the shared upregulated **(B)** and downregulated **(C)** genes in each RCC cell line analyzed and highlights the genes shared by all the cell lines. **(D)** qRT-PCR analysis of SIGIRR mRNA expression in normal kidney tissue (n = 13) compared to ccRCC patients’ sample (n = 21) **p < 0.01. Data are expressed as mean ± SEM relative to 18S expression. **(E)** qRT-PCR analysis of SIGIRR mRNA expression in RCC cell lines, in normal epithelial kidney cell line N2, and in platelets used as positive control. ***p<0,001; ****p < 0.0001. Data are expressed as mean ± SEM relative to GAPDH expression. **(F)** Micrograph of IHC performed using an antibody specific for SIGIRR (long isoform). One representative clear cell carcinoma sample is shown compared to normal kidney tissue. **(G)** Western blot analysis of SIGIRR expression on whole-cell extracts of A498, CAKI1, CAKI2, ACHN RCC cell lines, and platelet extract. B-Actin was used as loading control.

To profile the expression levels of mRNA encoding for Toll-like receptors (TLR), interleukin-1 (IL1) receptor family members, and their corresponding signaling mediators in kidney cancer, we performed a real-time PCR (RT-PCR) array analysis. Specifically, we screened the expression levels of 84 mRNAs together with seven housekeeping genes in a small cohort of 12 clear cell RCC (ccRCC) patient samples as compared to their coupled adjacent normal tissues (see **Supplementary Table 1** for a list of the genes analyzed). From this screening, seven genes emerged as differentially expressed in the malignant tissue (**Figure 1A**); in detail, TLR3 (toll-like receptor 3) was the only one upregulated while six transcripts were downregulated. Among the most downregulated molecules, SIGIRR (single immunoglobulin domain-containing IL1R-related protein), TOLLIP (tool interacting protein), and PTGS2 (prostaglandin-endoperoxide synthase 2, known also as COX2) emerged as significantly lower in tumor samples, as well as PPARA (peroxisome proliferator activated receptor gamma), MAPK8 (mitogen-activated protein kinase 8), and NR2C2 (nuclear receptor subfamily 2 group C member 2).

Next, we analyzed the TLR pathway in different cell lines representative of different kidney tumor types: A498 (clear cell RCC, VHL mutated), CAKI1 [clear cell RCC, metastatic, VHL wt), CAKI2 (initially defined as ccRCC VHL wt, but recent data suggest that it is a primary papillary RCC cell line (41)], and ACHN (papillary RCC, metastatic, VHL wt). The same analysis was performed in the normal kidney epithelial cells N2, and the FD of gene expression in each RCC cell line versus renal cells was calculated (**Supplementary Figure S1**). Lower levels of SIGIRR and of TOLLIP, NFRKB, TNFRSF1A, and CD14 and of two distinct MAP kinases were detected in all the cancer cell lines analyzed (**Figure 1B**); on the contrary, IRAK1 and HSPD1 mRNAs were upregulated in all the tumor cells (**Figure 1C**).

Bioinformatics analysis using the Oncomine resource confirmed downregulation of SIGIRR mRNA in different datasets of clear cell RCC compared to their normal counterpart (**Supplementary Figure S2**). Moreover, a strong SIGIRR protein expression in the kidney was reported in the Human Protein Atlas Database, while it was downregulated in RCC (https://www.proteinatlas.org/ENSG00000185187-SIGIRR/tissue/kidney).

Overall, our data demonstrate that renal epithelial cancer tissues and cell lines show a specific and distinctive pattern of inflammatory molecule expression marked by a consistent SIGIRR mRNA downregulation.

### SIGIRR Is Markedly Downregulated in Clear Cell Renal Cell Carcinoma

To validate all these observations, we measured by RT-PCR the mRNA levels of protein-coding SIGIRR isoforms (201-202-203-220) in a total of 20 primary ccRCC samples (including the 12 array samples) and 13 normal kidney tissues; we confirmed significantly lower levels of SIGIRR mRNA in ccRCC as compared to the normal epithelium (**Figure 1D**). However, since SIGIRR is expressed by several cell types, including tumor-infiltrating leukocytes, it would be important to understand which cell types, in addition to cancer cells, express SIGIRR.

We initially analyzed bioinformatic data from the Human Protein Atlas that showed high SIGIRR expression in normal kidney tubular epithelial cells and protein downregulation in clear cell carcinoma (link to websites: https://www.proteinatlas.org/ENSG00000185187-SIGIRR/tissue/kidney; https://www.proteinatlas.org/ENSG00000185187-SIGIRR/pathology/renal+cancer). Next, by taking advantage of an antibody developed in our lab and directed against a long isoform (34), we analyzed the SIGIRR protein expression in ccRCC tissues by immunohistochemistry (IHC). We confirmed that SIGIRR protein is highly expressed in normal tissue (n = 5), specifically within epithelial cells while ccRCC (n = 12) expressed low to undetectable levels of SIGIRR (**Figure 1F**).

Similarly, we confirmed the same downregulation of SIGIRR mRNA (protein-coding isoforms 201, 202, 203, 220, 218, 211) and protein levels in all the RCC cell lines in comparison to N2 normal kidney cells, as assessed by RT-PCR and Western blot analysis, respectively [**Figures 1E, G** where platelets acted as internal positive control (42)]. In the Western blot, the antibody detects different bands. SIGIRR glycosylations were previously reported in addition to alternative splicing (34, 43); all these variants may contribute to the different bands observed in the Western blot as previously reported. Unglycosylated SIGIRR is expected around 46/55 kDa while glycosylated SIGIRR around 65–90 kDa; however, from Western blot analysis we cannot assign each band to a specific splicing or glycosylated isoform.

To address the possible role of other cell types contributing SIGIRR mRNA expression, we analyzed data from scRNA-seq studies that are available at the Human Protein Atlas Database. We confirmed that SIGIRR is part of a cluster assigned to “Proximal tubular cells - Tubular reabsorption” with high confidence (link: https://www.proteinatlas.org/ENSG00000185187-SIGIRR/single+cell+type). Moreover, in the kidney, SIGIRR mRNA is more expressed in epithelial cells as compared to immune cells (link to website at https://www.proteinatlas.org/ENSG00000185187-SIGIRR/single+cell+type/kidney).

**Figure 2:**
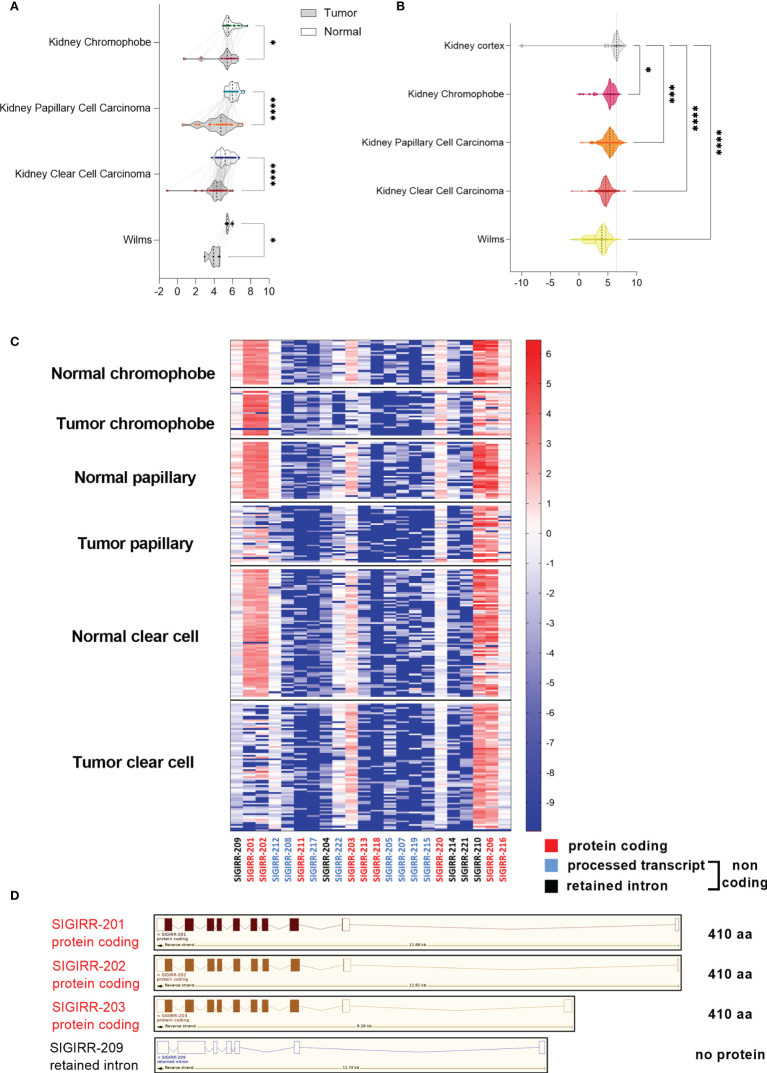
SIGIRR is downregulated in kidney cancer. **(A)** Bioinformatic analysis of transcriptomic profiles in different kidney tumor histotypes compared with their adjacent non-tumor tissue was performed with Xena Browser (data from TCGA database). SIGIRR expression levels are reported in chromophobe (n = 25), papillary (n = 32), clear cell carcinoma (n = 72), and Wilms tumor (n = 5) versus their non-tumor counterpart., *p<0,05; ****p<0,0001 **(B)** RNA-seq data relative to the SIGIRR expression level in chromophobe (n = 66), papillary (n = 32), clear cell carcinoma (n = 531), and Wilms tumor (n = 126) versus normal kidney cortex (n = 28). RSEM-normalized expression levels are represented with the violin plot, *p<0,05; ***p<0,001; ****p<0,0001. **(C)** Heatmap representation of the difference between SIGIRR isoforms’ expression in chromophobe, papillary, and clear cell carcinoma versus their non tumor counterpart. Red labeling refers to protein coding isoforms, blue to processed transcript, and black to retained intron isoforms. **(D)** Schematic representation of SIGIRR human gene and isoforms (ENSG00000185187; www.ensembl.org). Only the most differentially expressed isoforms are depicted.

Because of the marked downregulation of SIGIRR in the cell lines originally derived from different RCC subtypes, we asked if SIGIRR was differentially expressed in clear cell versus other RCC histotypes. A specific bioinformatic analysis was performed using the Xena functional genomics explorer platform (https://xenabrowser.net) that retrieves the data from The Cancer Genome Atlas database TCGA including RNA-seq expression data in different RCC histotypes each associated with their non-tumor normal counterpart. A marked downregulation of SIGIRR mRNA levels was observed in ccRCC (n = 72 and p ≤ 0.0001) and in papillary cell carcinoma (n = 32 and p ≤ 0,0001); chromophobe tumor samples and Wilms tumor also expressed lower SIGIRR transcripts compared to their normal counterpart (**Figure 2A**; n = 25 and n = 5, respectively, p < 0.05). Next we compared all the different tumor types with the normal kidney cortex (mean ± SEM = 5.862 ± 0.599; n = 28) using the same browser and including 66 cases of chromophobe, 289 papillary, 531 clear cell, and 126 Wilms tumor; again, we detected a small downregulation in the chromophobe subtype (mean ± SEM = 5.185 ± 0.155; p = 0.0180) and a significant downregulation in papillary (mean ± SEM = 5.230 ± 0.063; p = 0,0003), clear cell RCC (mean ± SEM = 4.612 ± 0.035; p < 0.0001), and Wilms (mean ± SEM = 3.576 ± 0.126; p < 0.0001). Comparing the expression among the diverse histotypes, we observed lower levels in Wilms tumor and ccRCC versus all the other tumors (p < 0.0001) and no significant expression difference between papillary RCC and chromophobe (**Figure 2B**).

Next, looking for the mechanism regulating SIGIRR expression, we analyzed the methylation profiles of the genomic region around the human SIGIRR gene in the same TCGA datasets by using the Xena browser; no evident correlation between downregulation and methylation emerged (**Supplementary Figure S3**).

To assess if lower levels of SIGIRR mRNA correlated with disease progression and overall survival in 441 patients affected by RCC, the Kaplan–Meier survival curve of kidney cancer patients was analyzed for two groups of cases characterized by high or low SIGIRR mRNA either above or below the 75 or 25 quartiles, respectively. No significant difference was observed although a trend of shorter survival correlated with low SIGIRR levels (p-value = 0.1035, log-rank test statistic = 2.650; **Supplementary Figure S4A**). Specifically, analyzing clear cell, papillary, and chromophobe cases independently, no difference was observed based on SIGIRR expression (25 and 75 quartiles: **Supplementary Figures S4B–D**).

The SIGIRR gene is transcribed into 22 splice variants that include 9 protein coding and 13 variants non-protein coding: 8 processed transcripts and 5 retained intron isoforms. To assess whether SIGIRR downregulation in RCC could be due to differential isoform expression, we performed bioinformatic analysis with Xena on the basis of RNA-seq data of transcript-specific expression for “tumor samples” and normal adjacent tissue (TCGA data). Transcripts SIGIRR-201 and SIGIRR-202 (protein-coding isoforms) are not expressed in clear cell RCC and less expressed in papillary RCC, whereas in chromophobe samples the expression is similar between normal and tumoral tissues. Instead, isoform 203 (protein coding isoform) is expressed in normal coupled kidney tissue and downregulated in chromophobe and papillary but not in ccRCC (**Figures 2C, D**).

To address the contribution of SIGIRR-03 compared to SIGIRR-01 and SIGIRR-02, we performed the analysis with Xena’s Transcript View (https://ucsc-xena.gitbook.io/project/overview-of-features/transcript-view/) (44). The data in **Supplementary Figure S5** show that the SIGIRR-03 isoform contributes with a percentage around 20% of all the isoforms in both normal and tumor samples where it is not significantly downregulated. Other aberrant isoforms are more expressed in both normal and tumor samples but not functional as lacking 5′ exons (e.g., SIGIRR-210, SIGIRR-206, and SIGIRR-216).

While the inhibitory receptor SIGIRR is shut down in tumor cells, the expression of the activatory IL1R1 is maintained (data not shown), and we next asked if SIGIRR downregulation may unleash intrinsic IL1 signaling in RCC cells.

### Interleukin-1 Stimulation of RCC Cells Highlights an Intrinsic Signature of Inflammatory Pathway Activation

IL1 is responsible for the induction and autocrine production of IL6 in RCC cells (45–47); moreover, IL1 can target distinct RCC cell lines *in vitro* to induce NF-kB activation, metalloprotease (MMP) production, and cell adhesion molecule (e.g., ICAM1) expression favoring proliferation and invasiveness (48–50). However, the whole molecular pathways acting on cancer cells after IL1 stimulation are ill defined.

**Figure 3:**
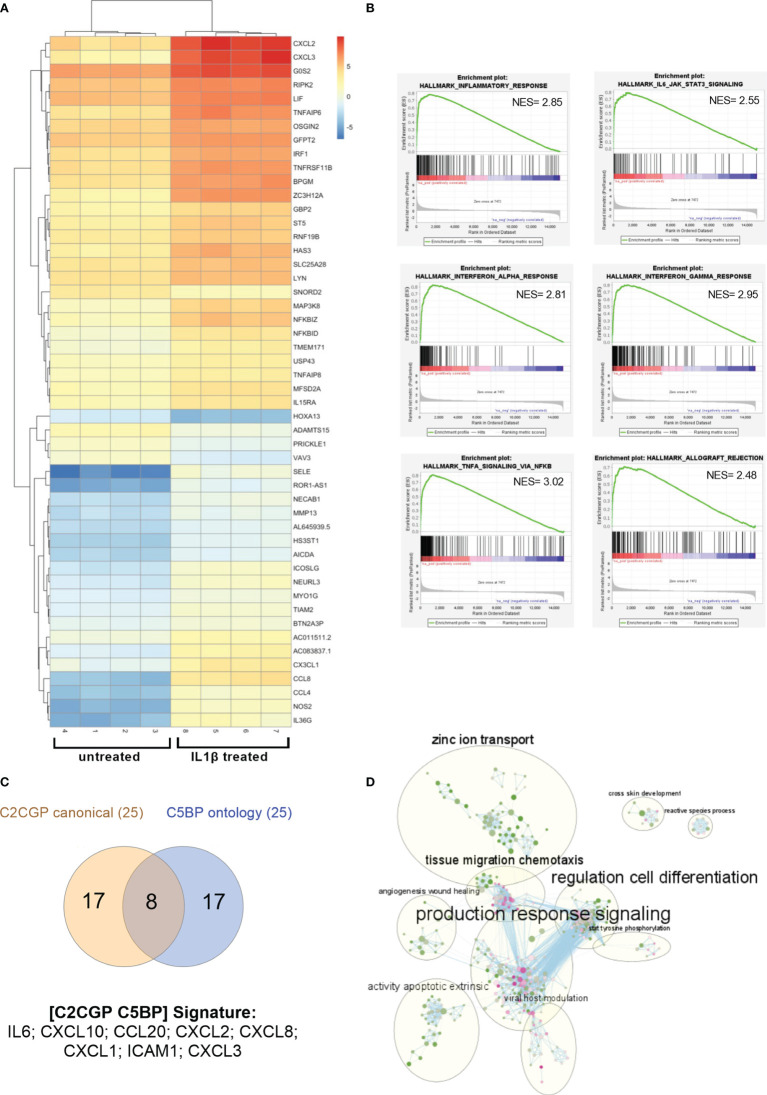
Gene expression profile in RCC cells after IL1 stimulation. **(A)** Heat map showing the clustered expression values of top 50 DEGs between treated with IL1β and control groups of A498 RCC cells. Genes are flagged as “differentially expressed” if they satisfy both the following conditions: nominal p-value < 0.01 and LogFC >1. Values have been scaled on rows. Color key is displayed on the right of the heat map. **(B)** Enrichment plots for selected hallmark gene sets found positively enriched in A498 treated with IL1β. The gene set name is shown at the top of the enrichment plot, the green curve represents the running enrichment score, and the black bars indicate the positions of the gene set hits on the rank ordered list in GSEA. The highest signal-to-noise values are indicated with red color, and blue indicates the lowest ones. **(C)** Venn diagram shows the shared upregulated genes obtained from leading edge analysis with GOBP and canonical pathways. **(D)** Enrichment map of GOBP enrichment analysis. The dots represent the node. The color and the size of the dots indicate respectively the NES values (from green 1.53 to pink 2.82) and the overlap size. The lines represent the link between nodes. The circles indicate the macro areas of similarity between nodes determined by AutoAnnotate. All the clusters with more than nine nodes are represented (except for “glial population glycogenesis” cluster with 10 nodes, not included here).

To characterize the genes regulated by IL1 in renal cancer cells, we performed RNA-sequencing of A498 cells either untreated or treated with interleukin-1β (IL1β; 30 ng/ml). From the analysis of all the replicates (n = 4), we found a total of 553 genes differentially expressed: 389 genes were upregulated while 164 were downregulated (**Figure 3A** shows the 50 most differentially expressed mRNAs). Among them, the atypical transcription factor, NF-kappaB inhibitor zeta (NFKBIZ) that is selectively induced by TLR and IL1R ligands, emerged as highly upregulated. To note, the transcripts encoding for the checkpoint ligand PD-L1 (CD274) were significantly upregulated after IL1 stimulation (LogFC 2,3; p-value 0.00082).

Upregulated genes (LogFC ≥1; p value <0.001) were analyzed with Enrichr, and we focused on BioPlanet 2019 and KEGG 2021 human-curated collections. As expected, we retrieved IL1 regulation of the extracellular matrix, binding of chemokines to chemokine receptors, and NF-kB canonical and alternative signaling pathways (**Supplementary Table 2**). Relevant to the kidney context, TWEAK regulation of gene expression (51) and PDL1 expression and PD1 checkpoint in cancer pathway were both enriched (p-value = 1.53E-04; combined score = 126.74). To identify potential transcription factors (TF) regulating the observed differential expression induced by IL1β, we screened the results against the collections related to TF analysis: the tumor-suppressor TP53 and the related protein TP63 emerged together with the expected NFkB family members (not shown).

To analyze the global perturbation of IL1β stimulation on the A498 RCC cell line, we performed Gene Set Enrichment Analysis (GSEA) on the transcriptional profiles against the hallmark gene sets. Among the most enriched pathways (NES >2 and FDR < 0.25), several immune-related signatures emerged such as TNFα signaling, interferon and inflammatory response, IL6-JAK-STAT3 signaling, and allograft rejection (**Figure 3B**).

Next, we performed GSEA against the gene ontology biological processes (GOBP) and the curated canonical pathway (C2CGP) collections of datasets; specifically, to determine which subsets of genes contributed the most to the enrichment signals, we performed the leading-edge analysis for the most enriched pathways. The intersection between leading edge analysis of GOBP and C2CGP showed distinct common upregulated genes including several chemokines, IL6 and ICAM1 (**Figure 3C**). In detail, a signature of eight genes, namely, IL6, CXCL10, CCL20, CXCL2, CXCL8, CXCL1, ICAM1, and CXCL3, emerged and highlighted ccRCC patients with adverse clinical outcomes expressing elevated levels of at least one of these mRNAs (**Supplementary Figure S6**). Specifically, the median month overall survival (95% CI) was 53.42 for the altered group (n = 92) and NA for the unaltered group (n = 418).

Finally, to address if besides the inflammatory and immunological pathways other molecular interactions or biological pathways were relevant, we built the EnrichmentMap on Cytoscape using the default parameters for similarity based on our GSEA of the GOBP pathways. We then looked for macroareas taking advantage of AutoAnnotate, an app that automatically groups and collapses clusters. In accordance with our previous data, the most overrepresented area is related to “production response signaling” which includes several inflammatory and immune-related pathways (**Figure 3D**). Notably, additional clusters emerged as composed of pathways involved in cell differentiation, tissue migration chemotaxis, zinc ion transport, apoptosis, angiogenesis, and wound healing (**Figure 3D**).

**Figure 4:**
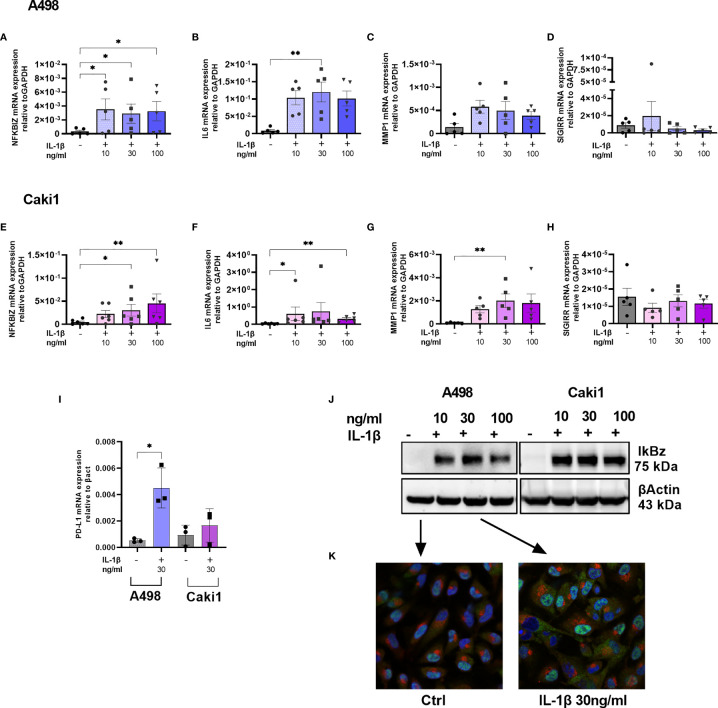
IL1-mediated cell activation and immune evasion in RCC. NFKBIZ **(A, E)**, IL6 **(B, F)**, MMP1 **(C, G)**, SIGIRR **(D, H)**, and PDL1 **(I)** mRNA expression levels in A498 **(A–D, I)** and CAKI1 **(E–H, I)** cell lines with or without stimulation with increasing concentration of IL1β (10–30–100 ng/ml) for 4 h were measured by qRT-PCR and expressed as mean ± SEM of at least five independent experiments. *p < 0.05, **p < 0.01. **(J)** IkBz protein expression after IL1β stimulation for 4 h in A498, and CAKI1 was assessed by Western blot analysis. B-Actin was used as loading control. **(K)** Representative confocal microscopy images of untreated and treated A498 cells with IL1β 30 ng/ml. IkBz protein in green, golgin in red, and 4,6-diamidino-2-phenylindole (DAPI) staining cell nuclei in blue (original magnification ×40).

To validate some of the differentially expressed genes identified, we focused our attention on two different ccRCC cell lines (Caki1 and A498); we stimulated the cells with increasing amounts of IL1β for 4 h, and we measured the expression levels of NFKBIZ as the most distinctive transcription factor of the IL1R and TLR signaling pathways that emerged from RNA-sequencing. We detected significantly increased amounts of NFKBIZ transcripts in both A498 and Caki1 cells (**Figures 4A, E**); this was paralleled by a corresponding increase of the mRNA encoding for the proinflammatory cytokine IL6 that is specifically controlled by NFKBIZ itself in a feedforward regulatory network and that emerged from RNA-sequencing as well (**Figures 4B, F**). Similarly, the mRNA specific for metalloprotease-1 (MMP-1) showed a trend of increase (**Figures 4C, G**) while SIGIRR mRNA levels were not affected (**Figures 4D, H**). The checkpoint protein PD-L1 was significantly increased in both A498 and Caki1 cells (**Figure 4I**). Somewhat surprisingly, the levels of the genes analyzed were not increased in a dose-dependent manner, suggesting that even low doses of IL1 are enough to reach full activation.

To validate IL1β-induced IkBz expression at the protein level, we performed Western blot analysis on Caki1 and A498 cell extracts; while untreated cells did not express IkBz, we detected increased levels after IL1β stimulation (**Figure 4J**). We performed an immunofluorescence experiment using antibodies specific for IkBz protein (green color) or for golgin (red color), and we stained the nuclei with DAPI (blue color); after stimulation, IkBz localized in the nucleus, but there was a bright and diffuse signal also in the cytoplasm with no signal in the Golgi (**Figure 4K**).

### IL1β-Mediated Signaling Is Dampened by SIGIRR Overexpression

**Figure 5:**
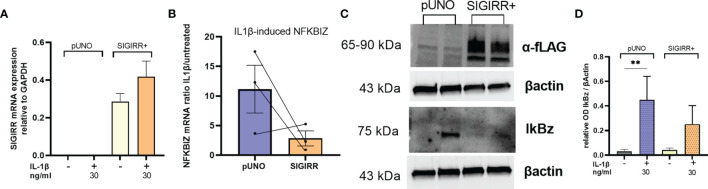
SIGIRR overexpression downregulates IL1 signaling in RCC cells. SIGIRR containing plasmid or empty vector were transiently transfected into A498 cells to induce SIGIRR overexpression **(A)**; transfected cells were analyzed for their responsiveness to IL1β (30 ng/ml) in terms of NFKBIZ **(B)** mRNA levels relative to GAPDH and B-actin expressed as ratio of IL1-treated/untreated (mean ± SEM of three independent experiments). **(C)** Western blot analysis of one representative experiment to analyze SIGIRR overexpression and IkBz regulation before and after IL1 stimulation. **(D)** Densitometric analysis of Western blot results (mean ± SEM of four independent experiments); **p<0,005.

To test if SIGIRR overexpression was able to dampen intrinsic IL1 signaling in RCC cells, we transiently transfected A498 with SIGIRR expression plasmid or empty vector; as expected, SIGIRR-transfected cells showed very high levels of SIGIRR mRNA and protein (**Figures 5A, C**). However, the transient transfection per se, either with the mock and even with SIGIRR-containing plasmid, caused an “aspecific” increase in the basal levels of expression of downstream mRNAs. Nonetheless, when we exposed the cells to IL1β for 4 h, we confirmed induction of NFKBIZ (**Figure 5B**) and IL6, PD-L1, and ICAM1 mRNAs (**Supplementary Figure S7**). A trend of lower induction was observed for all these genes when SIGIRR was overexpressed as compared to the empty vector: **Figure 5B** and **Supplementary Figure S7** show the average expression levels as the ratio of IL1-treated/untreated cells. Western blot analysis confirmed a decrease in IkBz protein levels in cells overexpressing SIGIRR as compared to mock transfected cells (**Figure 5D** shows one representative experiment out of 4).

Overall, these results suggest that SIGIRR downregulation unleashes IL1 signaling intrinsic to renal cell carcinoma cells.

## Discussion

To address if distinct immune receptors may be expressed by renal epithelial tumor cells and may directly regulate their pathobiology when associated with inflammation, we performed an expression profile of inflammatory receptors in kidney tumor tissues as compared to normal adjacent samples. Distinct TLRs are expressed by RCC, but TLR3 is the only one significantly unregulated; accordingly, a previous report suggested that TLR3 may be exploited as a novel therapeutic target (52).

TLR2 and TLR4 are downregulated in three cell lines out of four analyzed; in contrast, TLR4 is upregulated in RCC tissue samples isolated from patients while TLR2 is slightly downregulated (**Supplementary Table 1**). NR2C2, MAPK8, PPARA, and PTGS2 are all downregulated in tumor versus normal tissues. NR2C2, nuclear receptor subfamily 2 group C member 2, encodes a protein that can either activate or repress gene transcription and plays a role in protecting cells from oxidative stress (https://www.genecards.org). MAPK8 is a member of the MAP kinase family involved in multiple processes such as proliferation, migration, and cell death and is a part of a cascade that regulates AP-1 transcriptional activity and NF-kB pathway. PPARA, peroxisome proliferator activated receptor alpha, is another nuclear transcriptional regulator involved in proliferation and inflammation and is antagonized by NR2C2. PTGS2, known as cyclooxygenase, is an inducible enzyme that regulates prostaglandin synthesis during inflammation. The overall functional role of these enzymes or transcription factors in respect to inflammatory stimulation will deserve further future investigation.

Finally, TOLLIP and SIGIRR, both negative regulators of inflammatory signaling, are downregulated in primary samples as well as cell lines.

While it was previously known that SIGIRR is highly expressed in the kidney (33), no data were available on the expression and function of this molecule in clear cell RCC. We herein demonstrate that SIGIRR is strikingly downregulated in RCC cells from both primary tumors and cell lines representing different tumor types. Bioinformatic analysis confirmed that SIGIRR mRNA levels are lowest in the most aggressive clear cell subtype of RCC, but it is downregulated also in the papillary and chromophobe tumors. A trend toward lower levels of SIGIRR mRNA expression was observed in patients with shorter overall survival in a large cohort of cases including all the different kidney cancer types from TCGA database (**Supplementary Figure S4**); however, when the analysis was performed by dividing the different tumor subtypes, no difference was observed among cases expressing different levels of SIGIRR, suggesting that SIGIRR downregulation specifically marks a clear cell RCC histotype (the most aggressive subtype) rather than overall survival.

It is not known if SIGIRR downregulation is a marker of the tumor cell of origin or if it is downregulated in tumor cells during/after carcinogenesis. While the exact origin of each tumor subtype is ill-defined, single-cell transcriptomics is shedding light on the cellular identity of renal tumors (53, 54); future studies will help to elucidate the exact molecular mechanisms governing SIGIRR expression in distinct RCC subtypes as compared to their “normal counterpart.”

The activation of TLR and IL1R family members is dampened by the SIGIRR receptor (28, 29). Accordingly, lack of SIGIRR in mouse models unleashes constitutive inflammation mainly mediated by macrophages and dendritic cells (55). Nonetheless, SIGIRR is highly expressed in NK cells where it represses their antitumor and antiviral activities acting as a checkpoint (56). On the other hand, tumor-suppressive functions of SIGIRR have been recently described in intestinal cancer where the exacerbated activation of epithelial cells and tumor-infiltrating leukocytes lacking SIGIRR favors cancer progression in different mouse models (30, 57).

Regarding the intrinsic role of SIGIRR to tumor cells, few studies are available. In chronic lymphocytic leukemia, SIGIRR is specifically downregulated at both RNA and protein levels (30, 31). Treatment with demethylating agents restores SIGIRR expression, suggesting that a mechanism of DNA methylation operating in tumor cells is responsible for SIGIRR shutdown; however, methylation profiles around SIGIRR were not different among normal and leukemic cells, suggesting that other regulatory mechanisms and/or genes are involved (30). Our current data on the methylation profile of ccRCC samples from TCGA database indicate no evident differential methylation around the SIGIRR promoter in tumor versus normal samples, again suggesting that additional mechanisms regulating SIGIRR are yet to be identified.

In addition, an epithelial intrinsic tumor-suppressive role of SIGIRR was demonstrated in mice where SIGIRR protected from genetically driven colon carcinogenesis (30). Quite the opposite, in breast cancer SIGIRR is upregulated and promotes tumor growth (32). To understand how the observed SIGIRR downregulation impacted on renal cancer cells, we first aimed at the identification of the key molecules induced by IL1 to address if they were modulated by SIGIRR.

We performed RNA-sequencing analysis of the A498 cell line either untreated or treated with IL1β *in vitro*. Overall, the data show an inflammatory signature similar to immune cells and characterized by prototypic molecules such as cytokines (e.g., IL6) and chemokines (e.g., IL8), checkpoint proteins (e.g., PD-L1), metalloproteases (e.g., MMP1), and transcription factors (e.g., NFKBIZ). Among them, a signature of eight genes including IL6, CXCL10, CCL12, CXCL12, CXCL8, CXCL1, ICAM1, and CXCL3 emerged and highlighted patients with adverse clinical outcomes (TCGA database, **Supplementary Figure S6**). In addition, pathways involved in cell differentiation, tissue migration chemotaxis, zinc ion transport, apoptosis, angiogenesis, and wound healing emerged and are potentially highly relevant in the context of cancer. Among the differentially expressed genes resulting from RNA-seq, the following were validated by real-time PCR and used as surrogate markers after IL1 stimulation in the final experiments where SIGIRR expression was manipulated: MMP1, ICAM1, PD-L1, IL6, and NFKBIZ.

The induction of MMP1 and ICAM was previously reported to be activated by IL1 in renal cancer cells, suggesting that this pathway could favor tumor metastasis (49, 50).

PD-L1 (CD274) expression has a prognostic value in RCC where a higher level of this immune checkpoint regulator is a negative prognostic factor (58, 59). Our data demonstrate that IL1b may directly upregulate PD-L1 in cancer cells, thus explaining a mechanistic way whereby inflammatory stimulation may favor immune escape while at the same time recruiting additional immune cells through chemokine release. Moreover, IL1-stimulated RCC cells expressed higher levels of CXCL8 (IL-8) that was previously associated with lack of response to immune checkpoint inhibitors (60), further reinforcing the pro-tumoral role of IL1 not only on the immune microenvironment but also acting directly on epithelial malignant cells.

IL6 is a well-known pro-tumoral autocrine factor for RCC cells (22, 45); moreover, a recent report demonstrated that epithelial memory is induced by inflammatory insults and that may promote pancreatic tumorigenesis mainly through the activity of immune-derived interleukin-6 directed onto cancer cells (61). It is tempting to speculate that a similar mechanism may operate in renal cancer whereby the production of interleukin-1 by tumor-infiltrating monocytes may not only stimulate RCC cells to sustain an inflammatory microenvironment but also induce epithelial memory impacting on tumor progression through IL6 production.

To note, besides the involvement of the classic NFKB pathway, RNA-seq data showed that IL1β stimulation led to a significant increase in NFKBIZ expression. NFKBIZ is an atypical transcription co-factor belonging to the IKB family specifically induced by TLR and IL1R (62); it is necessary to mount an efficient immune response to pattern recognition receptors specifically regulating the secondary wave of innate immunity after classic NF-kB activation (63). Moreover, its overexpression has been implicated in cell viability and chemoresistance in B-cell lymphomas (64, 65); however, no data were available on NFKBIZ in RCC. We show that NFKBIZ mRNA is low to undetectable in unstimulated tumor cells while it is induced by IL1β in two different ccRCC cell lines, although it was not increased in a dose-dependent manner. The corresponding IkBz protein is produced and localized mainly into the nucleus, marking ongoing inflammatory signaling. The cytoplasmatic form of IkBz that was detected in stimulated tumor cells suggests a novel function unrelated to DNA transcription to be further explored.

The overexpression of SIGIRR in RCC cells attenuated IL1-induced NFKBIZ activation, with a trend of effect on IL6, PD-L1, and ICAM1 induction, suggesting that SIGIRR manipulation may be beneficial to block IL1 in kidney cancer. Nevertheless, a possible limitation of our study is that transient transfection may underestimate the whole functional effects of SIGIRR overexpression, and future experiments are needed to dissect the molecular pathways involved downstream of SIGIRR upregulation. Moreover, we should not overlook the potential effect of other molecules in regulating IL-1-mediated effects; in particular, TOLLIP downregulation may contribute to the sensitivity of the cells to IL-1, and TLR3 overexpression may modulate inflammatory signaling as well.

IL1 treatment was initially proposed for immunotherapy of RCC either alone or in combination with IL2 with variable results (66–68); it was suspected that the toxicity associated with IL2 treatment could be partially due to the induction of IL1 and thus anti-IL1 molecules such as a soluble IL1R were tested in both preclinical and clinical studies with no clear protection from toxic effects (69).

Additional studies underlined a potential pro-tumoral and pro-metastatic role of IL1 acting on the endothelial cells and on the invasive potential of cancer cells (49, 70, 71); moreover, IL1-producing monocytes and macrophages or IL1-dependent myeloid-derived suppressor cells were scrutinized for their role in shaping a protumor immune microenvironment in mice (21, 27, 72). Anti-IL1 or anti-IL1R antibodies delayed tumor progression in different mouse models (27) (72). Based on all these observations, a clinical trial is ongoing with anti-IL1 antibodies together with immune checkpoint inhibitors (NCT04028245; link: https://clinicaltrials.gov/ct2/show/NCT04028245) (73).

In summary, we characterized the expression of SIGIRR and the activation of downstream pathways in RCC after IL1B stimulation. Our study shows that SIGIRR downregulation in RCC cells unleashes an inflammatory signaling intrinsic to renal cancer cells that leads to NFKBIZ activation, IL6 induction, MMP1 upregulation, and notably higher levels of PD-L1 and ICAM1 expression. Elucidating the molecular mechanisms leading to SIGIRR deregulation in RCC and targeting them may be crucial to dampening pro-tumoral pathways. In addition, manipulating SIGIRR expression in cancer cells may help to relieve IL1-mediated RCC activation and may complement the use of targeted drugs as well as checkpoint inhibitors; at the same time, a careful analysis of SIGIRR expression along markers of intrinsic cancer cell inflammation may help to dissect tumor heterogeneity as well as immunotherapy response, which is becoming pivotal in the clinical management of advanced and metastatic RCC.

## Data Availability Statement

The original contributions presented in the study are publicly available. This data can be found here: https://www.ncbi.nlm.nih.gov/geo/query/acc.cgi?acc=GSE205851.

## Ethics Statement

The studies involving human participants were reviewed and approved by the Institutional Ethics Committee of San Raffaele Scientific Institute (Milan, Italy). The patients/participants provided their written informed consent to participate in this study.

## Author Contributions

MEM contributed to the conception and design of the study, performed the experiments and bioinformatic and statistical analysis, and wrote sections of the manuscript. IS performed the bioinformatic and statistical analyses and wrote sections of the manuscript. MV performed the experiment. MR analyzed the RNA sequencing data and supervised the bioinformatic analyses. CD performed the histochemical analysis. AL and UC provided patient samples and clinical information, supervised the specific analysis, and revised the manuscript. MM designed the study, coordinated the research, and wrote the manuscript. All authors contributed to the article and approved the submitted version.

## Funding

This work was supported in part by AIRC (IG-13042 and IG-23088).

## Conflict of Interest

Author MV is employed by Engitix Therapeutics Ltd.

The remaining authors declare that the research was conducted in the absence of any commercial or financial relationships that could be construed as a potential conflict of interest.

## Publisher’s Note

All claims expressed in this article are solely those of the authors and do not necessarily represent those of their affiliated organizations, or those of the publisher, the editors and the reviewers. Any product that may be evaluated in this article, or claim that may be made by its manufacturer, is not guaranteed or endorsed by the publisher.
