# Supplementary material for: SIGIRR Downregulation and Interleukin-1 Signaling Intrinsic to Renal Cell Carcinoma
Source: Front Oncol. 2022 Jun 22;12:894413. doi: 10.3389/fonc.2022.894413 (PMC9256934; doi:10.3389/fonc.2022.894413)
Supplement: Supplementary file 1 [file DataSheet_1.pdf]

SUPPLEMENTARY FILES

Figure S1. Scatter plot showing array gene expression measured by **R**eal-Time PCR of RCC cell lines versus normal kidney cells. A498 (**A**), CAKI1 (**B**), CAKI2 (**C**) and ACHN (**D**) are compared to N2 cells. The upregulated genes are indicated in red and the downregulated ones in blu colour.

Figure S2. SIGIRR relative expression in normal versus RCC samples; data from previously published microarray analyses as indicated are reported for different probes (52940_at in panels A,C,E and 218921_at in panels B,D,F).

Figure S3. DNA methylation profile of SIGIRR in renal cancer and adiacent normal tissue: Heat map representing DNA methylation status of SIGIRR genomic region retrieved from TCGA (The Cancer Genome Atlas) PAN-CANCER database. Methylation levels (Illumina 450k arrays data) were retrieved for renal cancer samples (850 samples) with web based tool UCSC Xena (University of California Santa Cruz Xena) and divided for cancer subtypes (KIRP=papillary renal cancer; KIRC=clear cell renal carcinoma; KICH=chromophobe renal cancer).

Figure S4. Kaplan Meier survival curves of RCC patients with high and low SIGIRR expression levels (above or below the quartile values respectively); pan RCC (**A**), clear cell RCC (**B**), papillary (**C**) and chromophobe (**D**) data were analyzed. Data from Xena browser.

Figure S5. Expression and exon structure of SIGIRR isoforms in normal kidney from GTEx and Renal Clear Cell Carcinoma (RCC) tissues from TCGA-KIRC. Each row in visualization shows the percentage of each isoform expression on the total expression levels (left) and transcript structure (right). Purple density indicates percentage of log2(TPM) from GTEx normal kidney samples (28). Green density indicates those from TCGA-KIRC samples (531). Exon plot follows the same order as density plots, in light blue are represented the untranslated exons, in blue the translated ones. All plots are generated using UCSC Xena browser.

Figure S6. Analysis of the signature identified in Figure 3C in kidney renal cell carcinoma datasets (TCGA PanCancer Atlas). The expression of CCL20, CXCL10, CXCL1, CXCL2, CXCL3, ICAM1, IL6 mRNA was analyzed in clear cell renal cell carcinoma samples to identify the cases with high expression levels («altered» cases only are shown in Panel A). The cohort was divided in two groups: «Altered» and «Unaltered» based on the expression levels of at least one mRNA of the signature. Median months survival (overall survival, disease specific survival, progression free or disease free) were analyzed for the two groups (B). Overall survival curve is shown in panel C.
The heatmap represents gene expression of the following genes: CXCL8, CCL20, CXCL10, CXCL1, CXCL2, CXCL3, ICAM1, IL6 and SIGIRR. Raw counts data have been retrieved from GDC platform through TCGA-biolinks R Bioconductor library, filtered for presence of at least 1 count in at least 72 samples and normalized to base2 logarithmic cpm (lcpm). Standardized lcpm values of the signature genes through a set of 613 TCGA-KIRC project cases, 540 Primary Tumor samples, 1 Additional - New Primary and 72 Solid Tissue Normal. Columns have been ordered based on the sample type (i.e., "normal", "primary tumor") (D).

Figure S7. Transfected cells with SIGIRR containing plasmid or empty vector were analyzed for their responsiveness to IL1β (30 ng/ml) in terms of IL6 (A), PDL1 (B) and ICAM1 (C) mRNA levels relative to GAPDH and/or B-actin expressed as ratio of IL1-treated/untreated (mean ± SEM of 3 independent experiments).

Table S1. List of genes analyzed by Real-Time PCR array.

Table S2. Summary of changes in Kegg-2021 and Bioplanet-2019 genesets ranked for their combination score. The statistical significance was assessed at p-value <0,005.

Supplementary Figure S1

A

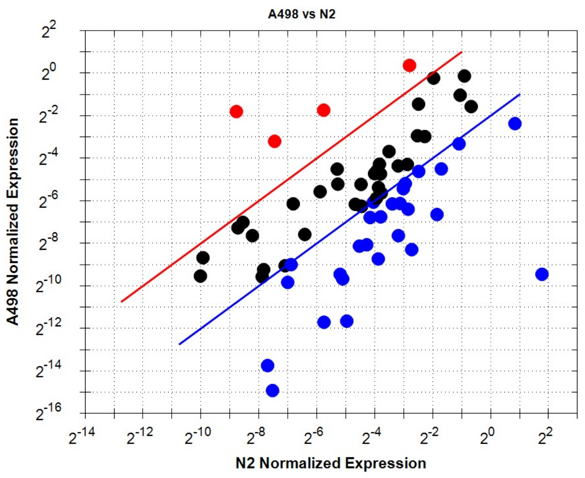

FOS
HSPD1
IRAK1
LY96

CD14
CLEC4E
CSF2
FADD
IKBKB
IL8
IRAK2
IRF3
MAP2K3

MAP3K7
MAPK8IP3
MYD88
NFKB1
NFKB2
NFKBIA
NFRKB
REL
RELA

SARM1
SIGIRR
TAB1
TICAM1
TIRAP
TLR4
TNFRSF1A
TOLLIP
TRAF6

B

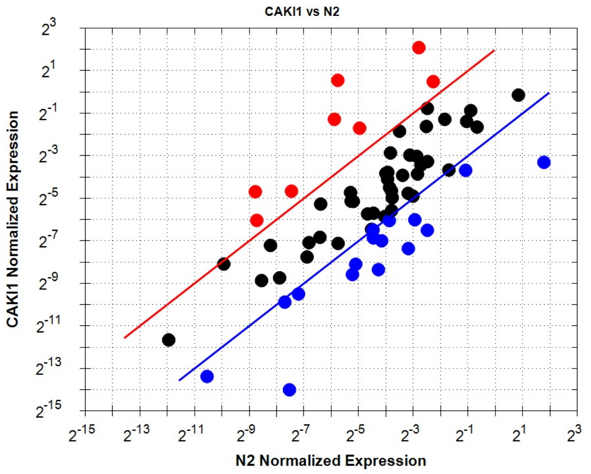

CSF2
FOS
HSPD1
IL12A
IL6
IRAK1
LY96
PRKRA

CD14
CLEC4E
IL1A
LTA
MAP2K3
MAPK8IP3
NFRKB
PELI1

PPARA
SIGIRR
TAB1
TLR2
TLR4
TNF
TNFRSF1A
TOLLIP

C

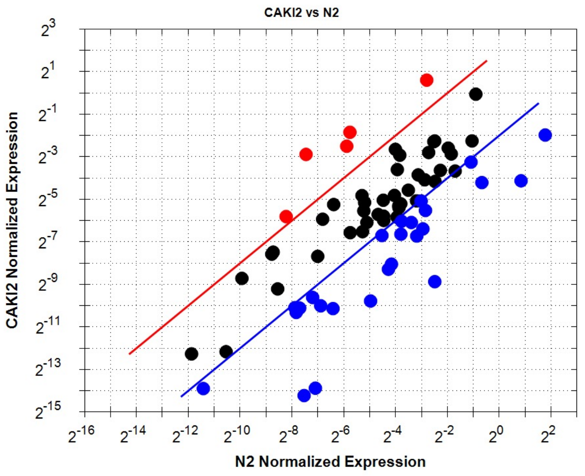

ECSIT
HSPD1
IRAK1
LY96
PRKRA

CD14
CLEC4E
CSF2
FADD
IKBKB
IL1A
IL1B
IL8

IRAK2
JUN
MAP2K3
MAPK8IP3
MYD88
NFRKB
NR2C2
PTGS2

SIGIRR
TICAM2
TIRAP
TLR10
TLR2
TLR5
TNFRSF1A
TOLLIP
TRAF6

D

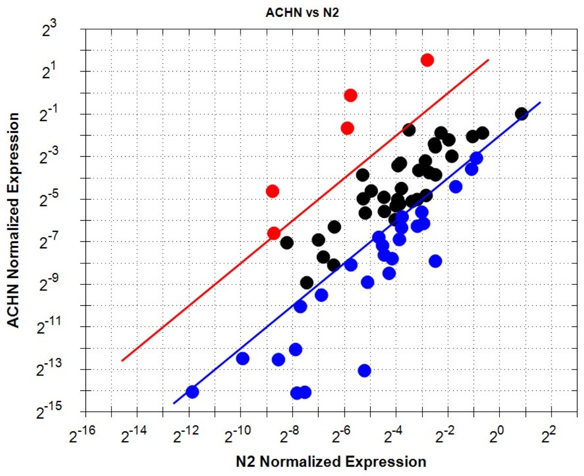

FOS
HSPD1
IL12A
IRAK1
PRKRA

BTK
CD14
CXCL10
ELK1
FADD
HSPA1A
IKBKB
MAP2K3
MAPK8IP3

MYD88
NFKB2
NFRKB
NR2C2
PPARA
PTGS2
SIGIRR
TICAM1
TIRAP

TLR1
TLR2
TLR4
TLR5
TNF
TNFRSF1A
TOLLIP
TAB1

Supplementary Figure S2

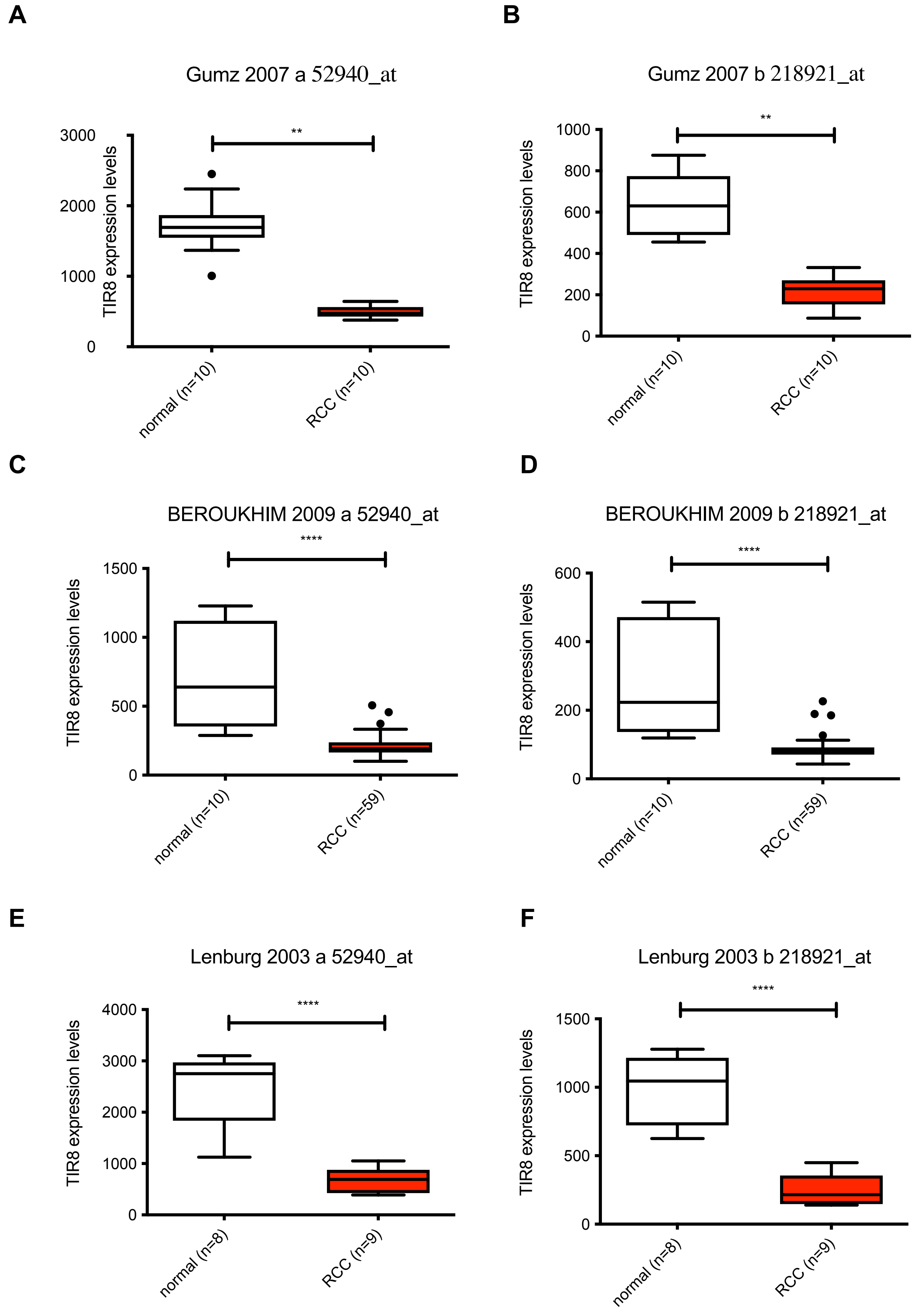

Supplementary Figure S3

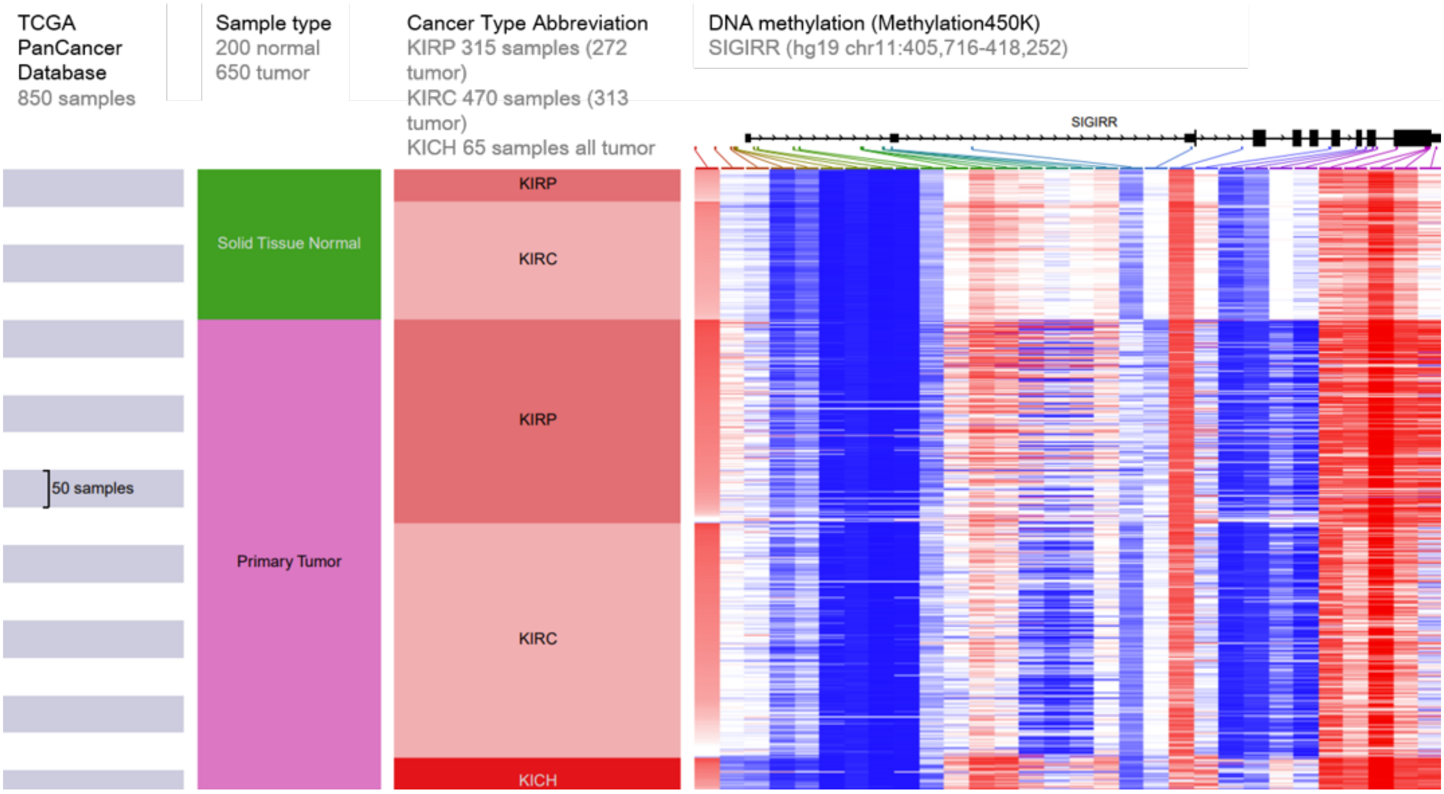

Supplementary Figure S4

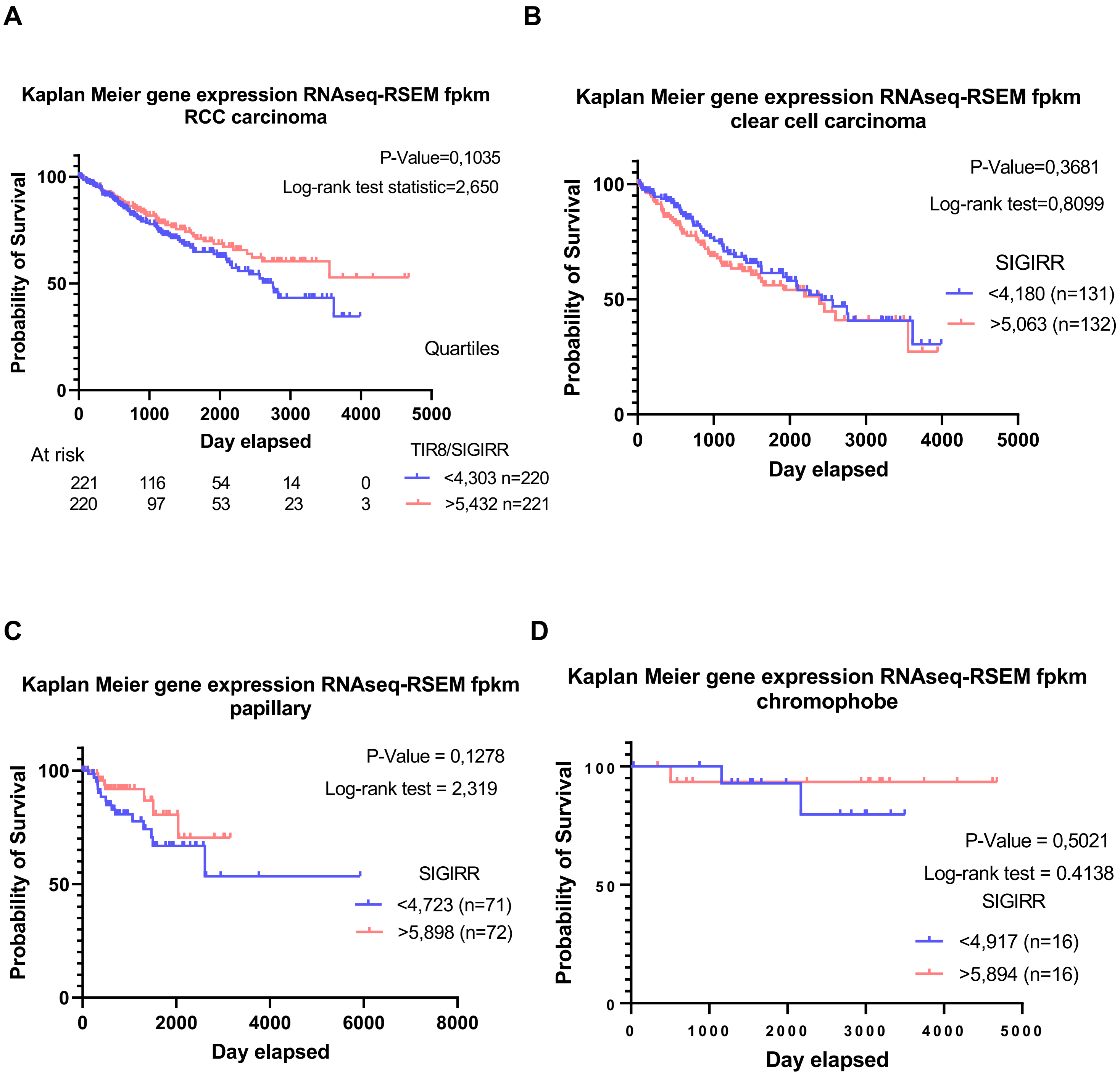

Supplementary Figure S5

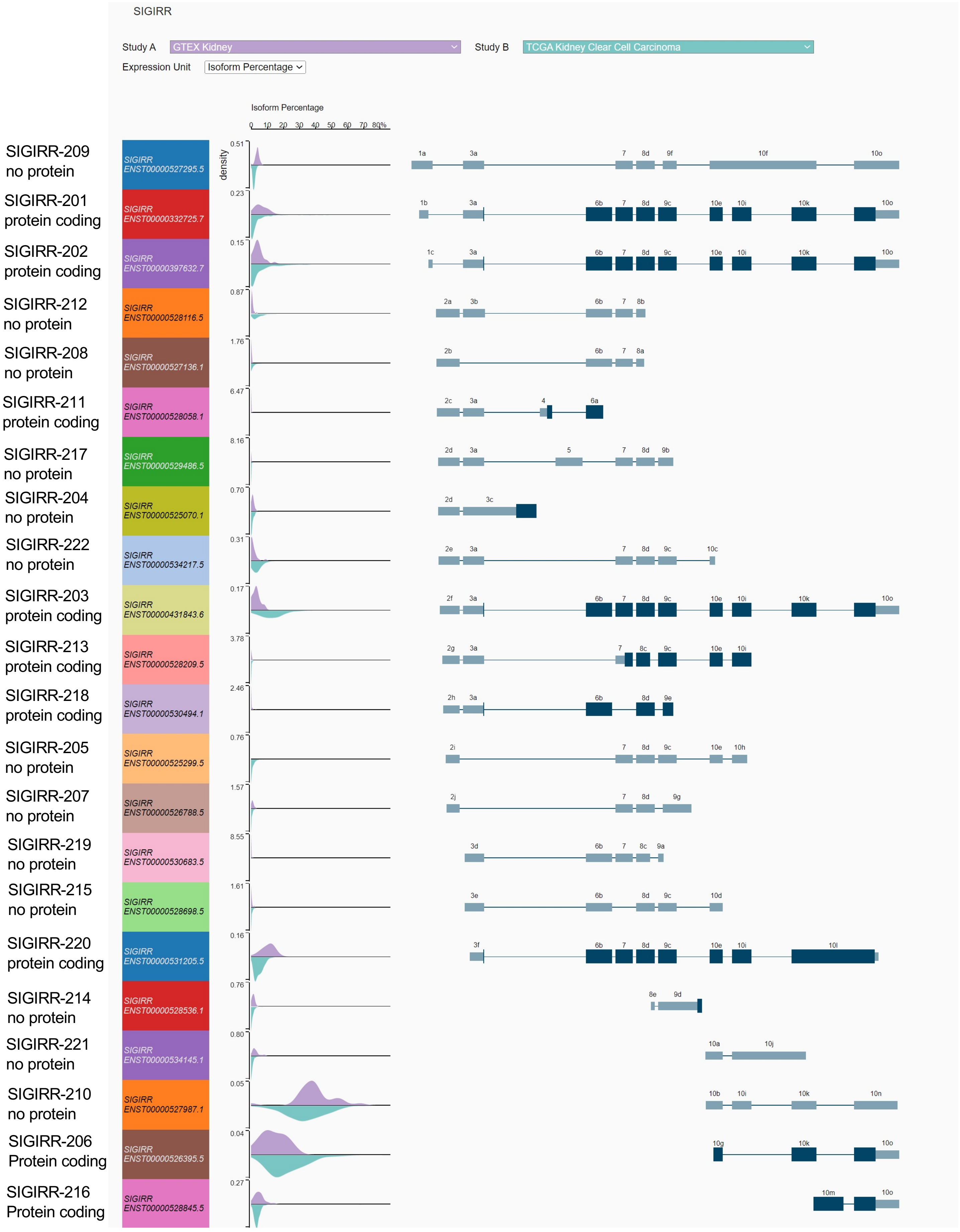

Supplementary Figure S6

A

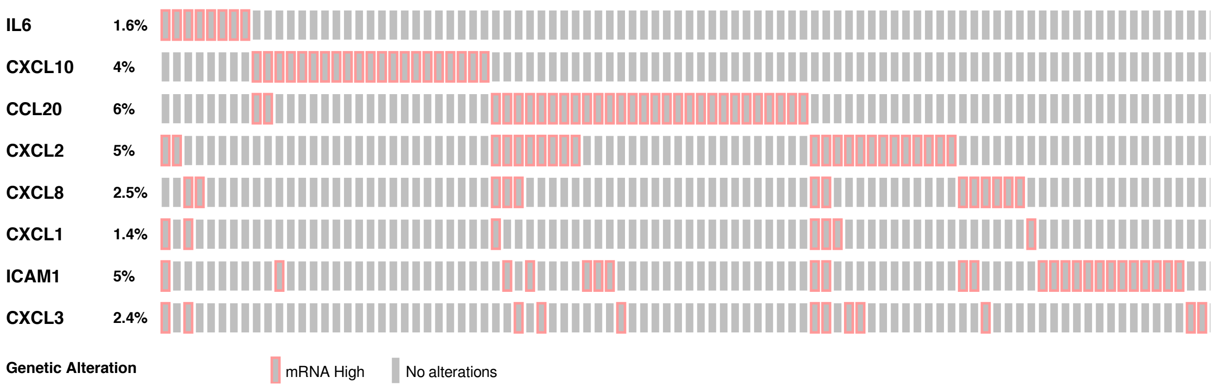

B

Survival Type	Number of Patients	# in Altered group	# in Unaltered group	Median months survival in Altered group (95% CI)	Median months survival in Unaltered group (95% CI)	p-Value	q-Value
Disease-specific	500	89	411	65.10 (52.21 - NA)	NA	1,16E-06	4,65E-06
Overall	510	92	418	53.42 (39.45 - 69.20)	NA	4,14E-06	8,27E-06
Progression Free	508	92	416	52.21 (31.50 - NA)	123.81 (89.88 - NA)	9,43E-04	1,26E-03
Disease Free	111	19	92	NA	NA	0,833	0,833

C

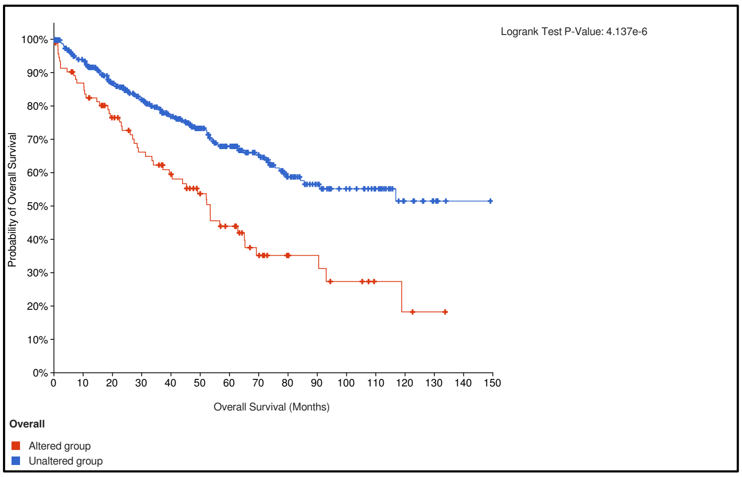

D

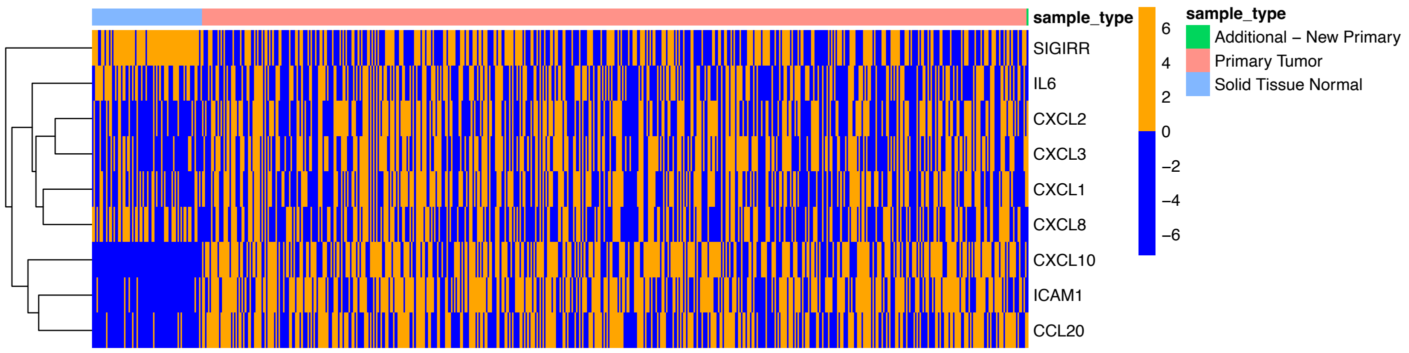

Supplementary Figure S7

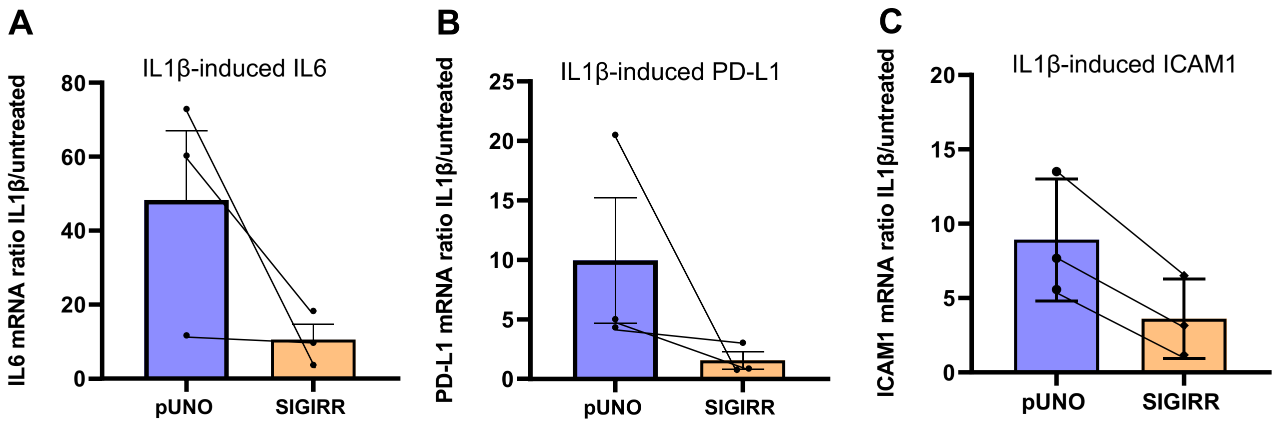

SUPPLEMENTARY TABLE 1 (Mantione et al)

SYMBOL	*FULL NAME*	*ENSEMBL GENE ID*	Average Normal 2^-Dct	Average Tumor 2^-Dct	FD	T- test
BTK	Bruton agammaglobulinemia tyrosine kinase	ENSG00000010671	0,014667856	0,017152351	1,169384	0,640921
CASP8	8, apoptosis-related cysteine peptidase	ENSG00000064012	0,094507392	0,106870767	1,130819	0,453518
CCL2	chemokine (C-C motif) ligand 2	ENSG00000108691	0,509442533	0,442566037	0,868726	0,383243
CD14	CD14 molecule	ENSG00000170458	0,09467089	0,158245057	1,671528	0,100765
CD180	CD180 molecule	ENSG00000134061	0,012771218	0,017766577	1,391142	0,537763
CD80	CD80 molecule	ENSG00000121594	0,011581017	0,017924593	1,547756	0,154287
CD86	CD 86 Molecule	ENSG00000114013	0,024712679	0,052659992	2,13089	0,019709
CHUK	conserved helix-loop-helix ubiquitous kinase	ENSG00000213341	0,131020834	0,07020759	0,535851	2,78E-05
CLEC4E	C-type lectin domain family 4, member E	ENSG00000166523	0,016565868	0,021395817	1,29156	0,352857
CSF2	colony stimulating factor 2 (granulocyte-macrophage)	ENSG00000164400	0,014971452	0,005526107	0,36911	0,03559
CSF3	colony stimulating factor 3 (granulocyte)	ENSG00000108342	0,005879927	0,001217911	0,20713	0,112683
CXCL10	chemokine (C-X-C motif) ligand 10	ENSG00000169245	0,023833133	0,166364846	6,980402	0,0815
ECSIT	ECSIT homolog (Drosophila)	ENSG00000130159	0,264283946	0,164526887	0,622538	0,140995
EIF2AK2	eukaryotic translation initiation factor 2-alpha kinase 2	ENSG00000055332	0,17502307	0,189310239	1,08163	0,466012
ELK1	ELK1, member of ETS oncogene family	ENSG00000126767	0,053667162	0,034871506	0,649774	0,013976
FADD	Fas (TNFRSF6)-associated via death domain	ENSG00000168040	0,11541371	0,063060921	0,54639	0,002019
FOS	FBJ murine osteosarcoma viral oncogene homolog	ENSG00000170345	3,343023636	1,499248051	0,448471	0,01925
HMGB1	high mobility group box 1	ENSG00000189403	0,098808851	0,127527455	1,290648	0,090715
HRAS	v-Ha-ras Harvey rat sarcoma viral oncogene homolog	ENSG00000174775	0,094245898	0,047822977	0,507428	4,96E-05
HSPA1A	heat shock 70kDa protein 1A	ENSG00000204389	2,878213089	0,759437992	0,263857	0,015574
HSPD1	heat shock 60kDa protein 1 (chaperonin)	ENSG00000144381	0,646146459	0,324285664	0,501876	0,00057
IFNA1	interferon, alpha 1	ENSG00000197919	0,007890498	0,002407228	0,305079	0,095908
IFNB1	interferon, beta 1, fibroblast	ENSG00000171855	0,006061831	0,001654289	0,272902	0,116085
IFNG	interferon, gamma	ENSG00000111537	0,005925827	0,006004656	1,013303	0,98423
IKBKB	inhibitor of kappa light polypeptide gene enhancer in B-cells, kinase beta	ENSG00000104365	0,103451617	0,069578442	0,67257	0,068345
IL10	interleukin 10	ENSG00000136634	0,011406072	0,007018263	0,615309	0,288807
IL12A	interleukin 12A (cytotoxic lymphocyte maturation factor 1)	ENSG00000168811	0,006962965	0,001926416	0,276666	0,103786
IL1A	interleukin 1, alpha	ENSG00000115008	0,006199424	0,001623182	0,261828	0,112697
IL1B	interleukin 1, beta	ENSG00000125538	0,066505141	0,028696839	0,431498	0,243767
IL2	interleukin 2	ENSG00000109471	0,008824891	0,001789415	0,202769	0,026853
IL6	interleukin 6 (interferon, beta 2)	ENSG00000136244	0,036984539	0,010682188	0,288828	0,04074
IL8	interleukin 8	ENSG00000169429	0,076442297	0,120802729	1,580313	0,451331
IRAK1	interleukin-1 receptor-associated kinase 1 ENSG00000184216	ENSG00000184216	0,112838554	0,108289019	0,959681	0,735196
IRAK2	interleukin-1 receptor-associated kinase 2	ENSG00000134070	0,032358209	0,024406783	0,754269	0,249447
IRAK4	interleukin-1 receptor-associated kinase 4	ENSG00000198001	0,08325583	0,093087094	1,118085	0,514529
IRF1	interferon regulatory factor 1	ENSG00000125347	0,218878777	0,224176405	1,024203	0,93797
IRF3	interferon regulatory factor 3	ENSG00000126456	0,061142819	0,046055549	0,753245	0,158863
JUN	jun proto-oncogene	ENSG00000177606	3,347015047	1,419268782	0,42404	0,055517
LTA	lymphotoxin alpha (TNF superfamily, member 1)	ENSG00000226979	0,006668161	0,003592634	0,538774	0,262064
LY86	lymphocyte antigen 86	ENSG00000112799	0,034723638	0,082509052	2,376164	0,023993
LY96	lymphocyte antigen 96	ENSG00000154589	0,024298019	0,070102295	2,885103	0,033221
MAP2K3	mitogen-activated protein kinase kinase 3	ENSG00000034152	0,191450515	0,107808051	0,563112	0,033543
MAP2K4	mitogen-activated protein kinase kinase 4	ENSG00000065559	0,125373718	0,117040585	0,933534	0,555873
MAP3K1	mitogen-activated protein kinase kinase kinase 1	ENSG00000095015	0,171379435	0,124615829	0,727134	0,133407
MAP3K7	mitogen-activated protein kinase kinase kinase 7	ENSG00000135341	0,135901163	0,110635617	0,814089	0,039993
MAP4K4	mitogen-activated protein kinase kinase kinase kinase 4	ENSG00000071054	0,173305321	0,212423318	1,225717	0,142226
MAPK8	mitogen-activated protein kinase 8	ENSG00000107643	0,279166423	0,111086085	0,397921	0,000523
MAPK8IP3	mitogen-activated protein kinase 8 interacting protein 3	ENSG00000138834	0,322108115	0,154905188	0,480911	0,024024
MYD88	myeloid differentiation primary response gene (88)	ENSG00000172936	0,123359866	0,081704153	0,662324	0,051078
NFKB1	nuclear factor of kappa light polypeptide gene enhancer in B-cells 1	ENSG00000109320	0,187700449	0,137303261	0,731502	0,030592
NFKB2	nuclear factor of kappa light polypeptide gene enhancer in B-cells 2 (p49/p100)	ENSG00000077150	0,22554343	0,201438353	0,893124	0,472422
NFKBIA	nuclear factor of kappa light polypeptide gene enhancer in B-cells inhibitor, alpha	ENSG00000100906	0,393462821	0,392094493	0,996522	0,987698
NFKBIL1	nuclear factor of kappa light polypeptide gene enhancer in B-cells inhibitor-like 1	ENSG00000204498	0,362948137	0,266617452	0,734588	0,338401
NFRKB	nuclear factor related to kappaB binding protein	ENSG00000170322	0,133152764	0,083590223	0,627777	0,000451
NR2C2	nuclear receptor subfamily 2, group C, member 2	ENSG00000177463	0,180161086	0,076763357	0,426082	2,13E-06
PELI1	pellino homolog 1 (Drosophila)	ENSG00000197329	0,139268538	0,15511009	1,113748	0,476131
PPARA	peroxisome proliferator-activated receptor alpha	ENSG00000186951	0,334457278	0,106275903	0,317756	3,64E-05
PRKRA	protein kinase, interferon-inducible double stranded RNA dependent activator	ENSG00000180228	0,1087809	0,072488076	0,666368	0,017615
PTGS2	prostaglandin-endoperoxide synthase 2	ENSG00000073756	0,057942239	0,01686891	0,291133	0,009693
REL	v-rel reticuloendotheliosis viral oncogene homolog (avian)	ENSG00000162924	0,116753048	0,079886099	0,684231	0,108758
RELA	v-rel reticuloendotheliosis viral oncogene homolog A (avian)	ENSG00000173039	0,289275175	0,24514619	0,84745	0,233663
RIPK2	receptor-interacting serine-threonine kinase 2	ENSG00000104312	0,056612712	0,061345861	1,083606	0,585193
SARM1	sterile alpha and TIR motif containing 1	ENSG00000004139	0,069410866	0,05398233	0,777722	0,148951
SIGIRR	single immunoglobulin and toll-interleukin 1 receptor (TIR) domain	ENSG00000185187	0,335240422	0,067603787	0,201658	0,000662
TAB1	TGF-beta activated kinase 1/MAP3K7 binding protein 1	ENSG00000100324	0,247204712	0,145345304	0,587955	0,004386
TBK1	TANK-binding kinase 1	ENSG00000183735	0,105977763	0,111610994	1,053155	0,547533
TICAM1	toll-like receptor adaptor molecule 1	ENSG00000127666	0,02830433	0,030118833	1,064107	0,637411
TICAM2	toll-like receptor adaptor molecule 2	ENSG00000243414	0,013918413	0,009596832	0,689506	0,488879
TIRAP	toll-interleukin 1 receptor (TIR) domain containing adaptor protein	ENSG00000150455	0,036265056	0,02789138	0,769098	0,221268
TLR1	toll-like receptor 1	ENSG00000174125	0,029032925	0,050241091	1,730487	0,100674
TLR2	toll-like receptor 2	ENSG00000137462	0,011943891	0,007566288	0,633486	0,229161
TLR3	toll-like receptor 3	ENSG00000164342	0,057214805	0,117533883	2,054256	0,035732
TLR4	toll-like receptor 4	ENSG00000136869	0,049565174	0,274372032	5,535581	0,000157
TLR5	toll-like receptor 5	ENSG00000187554	0,059830897	0,081484501	1,361913	0,207136
TLR6	toll-like receptor 6	ENSG00000174130	0,038378947	0,018581265	0,484153	0,062281
TLR7	toll-like receptor 7	ENSG00000196664	0,023825941	0,023434018	0,983551	0,960577
TLR8	toll-like receptor 8	ENSG00000101916	0,021140418	0,059085953	2,794928	0,020446
TLR9	toll-like receptor 9	ENSG00000239732	0,01090954	0,021855305	2,003321	0,083212
TLR10	toll-like receptor 10	ENSG00000174123	0,008313937	0,002505262	0,301333	0,046506
TNF	tumor necrosis factor	ENSG00000232810	0,019818142	0,01182203	0,596526	0,178611
TNFRSF1A	tumor necrosis factor receptor superfamily, member 1A	ENSG00000067182	0,468032847	0,510840636	1,091463	0,441781
TOLLIP	toll interacting protein	ENSG00000078902	0,31282928	0,127403608	0,407262	0,000812
TRAF6	TNF receptor-associated factor 6	ENSG00000175104	0,086916798	0,097148143	1,117714	0,429991
UBE2N	ubiquitin-conjugating enzyme E2N (UBC13 homolog, yeast)	ENSG00000177889	0,112778229	0,071529943	0,634253	0,002128

SUPPLEMENTARY TABLE 2 (Mantione et al)

PATHWAYS: Bio Planet 2019				
**Name**	**P-value**	**Adjusted P-value**	**Odds Ratio**	**Combined Score**
**Interleukin-1 regulation of extracellular matrix**	3,73E-37	2,79E-34	26,87	2253,56
TNF-alpha effects on cytokine activity, cell motility, and apoptosi	2,91E-36	1,09E-33	23,55	1926,61
**Binding of chemokines to chemokine receptors**	2,227E-19	2,34E-13	22,09	736,61
Interferon alpha/beta signaling	1,99E-17	2,34E-13	19,02	633,45
**Thymic stromal lymphopoietin (TSLP) pathway**	2,586E-17	3,63E-15	16,16	612,86
TWEAK regulation of gene expression	2,23E-15	5,18E-08	25,78	528,52
**Alternative NF-kappaB pathway**	3,32E-15	1,43E-03	50,80	450,85
Low-density lipoprotein (LDL) pathway during atherogenesis	5,22E-12	1,43E-03	50,80	450,85
**MSP/RON receptor signaling pathway**	1,249E-09	1,43E-03	50,80	450,85
NOD signaling pathway	1,40E-04	2,26E-12	14,15	436,82
**Interferon signaling**	2,586E-17	3,22E-15	10,30	393,36
Epstein-Barr virus LMP1 signaling	1,32E-07	2,82E-06	23,94	379,16
**Interleukin-5 regulation of apoptosis**	6,73335E-16	6,29E-14	10,68	373,10
Immune system signaling by interferons, interleukins, prolactin, a	2,23E-16	5,54E-14	8,09	347,66
**Toll-like receptor signaling pathway regulation**	5,22371E-15	3,25E-13	10,29	338,50
Inactivation of BCL-2 by BH3-only proteins	2,41E-04	2,17E-03	38,10	317,35
**Cytokine-cytokine receptor interaction**	1,99E-17	3,22E-15	7,74	297,76
Interferon-gamma signaling pathway	5,27E-12	2,62E-10	11,16	289,73
**Type II interferon signaling (interferon-gamma)**	2,477E-09	8,81E-08	14,60	289,40
Interleukin-23-mediated signaling events	2,79E-08	8,12E-07	16,56	288,12

PATHWAYS: KEGG 2021 human				
**Name**	**P-value**	**Adjusted P-value**	**Odds Ratio**	**Combined Score**
**TNF signaling pathway**	2,056E-23	4,87E-21	18,03	941,89
Viral protein interaction with cytokine and cytokine receptor	1,46E-18	1,16E-16	15,94	654,69
**NF-kappa B signaling pathway**	3,765E-18	2,23E-16	15,15	607,90
Toll-like receptor signaling pathway	5,64E-17	2,68E-15	14,28	534,14
**IL-17 signaling pathway**	2,507E-13	6,60E-12	12,47	361,84
Cytokine-cytokine receptor interaction	1,61E-19	1,91E-17	7,88	341,18
**Lipid and atherosclerosis**	2,158E-16	8,52E-15	8,41	303,47
Influenza A	4,55E-15	1,54E-13	9,09	300,31
**Legionellosis**	7,957E-10	1,57E-08	13,84	289,97
NOD-like receptor signaling pathway	1,53E-14	4,53E-13	8,57	272,48
**Malaria**	3,546E-08	4,20E-07	12,91	221,46
Rheumatoid arthritis	3,24E-10	6,99E-09	10,04	219,45
**RIG-I-like receptor signaling pathway**	9,534E-09	1,41E-07	10,73	198,18
Chagas disease	1,24E-09	2,06E-08	9	184,62
**Epstein-Barr virus infection**	1,069E-11	2,53E-10	6,82	172,34
Cytosolic DNA-sensing pathway	3,52E-07	3,21E-06	9,74	144,67
**African trypanosomiasis**	0,000006209	4,75E-05	11,96	143,40
PD-L1 expression and PD-1 checkpoint pathway in cancer	1,53E-04	1,51E-07	8,07	126,74
**Chemokine signaling pathway**	0,00000119	2,06E-08	6,13	125,88
Kaposi sarcoma-associated herpesvirus infection	1,30E-09	2,06E-08	6,09	124,58
